# Advances and challenges on the path toward the SDGs: subnational inequalities in Mexico, 1990–2017

**DOI:** 10.1136/bmjgh-2020-002382

**Published:** 2020-10-29

**Authors:** Juan Pablo Gutierrez, Marcela Agudelo-Botero, Sebastian Garcia-Saiso, Carolina Zepeda-Tena, Claudio Alberto Davila-Cervantes, Maria Cecilia Gonzalez-Robledo, Nancy Fullman, Christian Razo, Bernardo Hernández-Prado, Gabriel Martínez, Simón Barquera, Rafael Lozano

**Affiliations:** 1 Center for Policy, Population & Health Research, Universidad Nacional Autónoma de México, Coyoacan, Mexico; 2 Departamento de Población y Desarrollo, Facultad Latinoamericana de Ciencias Sociales Mexico, Mexico DF, Mexico; 3 Centro de Investigación en Sistemas de Salud, Instituto Nacional de Salud Pública, Cuernavaca, Mexico; 4 Institute for Health Metrics and Evaluation, University of Washington, Seattle, Washington, USA; 5 Departamento Académico de Economía, ITAM, Alvaro Obregon, Mexico; 6 National Institute of Public Health, Cuernavaca, Mexico

**Keywords:** health policy, public health

## Abstract

**Background:**

The sustainable development goals (SDGs) have generated momentum for global health, aligning efforts from governments and international organisations toward a set of goals that are expected to reflect improvements in life conditions across the globe. Mexico has huge social inequalities that can affect access to quality care and health outcomes. The objective of this study is to analyse inequalities among Mexico’s 32 states on the health-related SDG indicators (HRSDGIs) from 1990 to 2017.

**Methods:**

These analyses rely on the estimation of HRSDGIs as part of the Global Burden of Disease study 2017. We estimated the concentration index for 40+3 HRSDGI stratified by Socio-demographic Index and marginalisation index, and then for indicators where inequalities were identified, we ran decomposition analyses using structural variables such as gross domestic product per capita, poverty and health expenditure.

**Findings:**

Mexico has made progress on most HRSDGIs, but current trends in improvement do not appear to fast enough to meet 2030 targets. Out of 43 HRSDGIs, we identified evidence of inequality between Mexico’s states for 30 indicators; of those, 23 HRSDGIs were unequal distributed affecting states with lower development and seven affecting states with higher development. The decomposition analysis indicates that social determinants of health are major drivers of HRSDGI inequalities in Mexico.

**Interpretation:**

Modifying current trends for HRSDGIs will require subnational-level and national-level policy action, of which should be informed by the latest available data and monitoring on the health-related SDGs. The SDGs’ overarching objective of *leaving no-one behind* should be prioritised not only for individuals but also for communities and other subnational levels.

**Key questions**

**What is already known?**

- A key challenge for Mexico’s—and other similar countries—health system is inequity.
- While there is evident progress on most of sustainable development goal (SDG) health indicators in the average, gaps remain and for some indicators are even wider.
- In addition, at the country-level, it appears that the pace of progress for many health-related SDG indicators (HRSDGIs) lags behind the 2030 targets.

**What are the new findings?**

- At the state level in Mexico, the pace of progress and absolute gains toward the HRSDGIs were heterogeneous.
- For most health-related SDGs indicators there is a relevant degree of inequality in the country by socioeconomic conditions.
- Decomposition analyses indicate that inequalities on the HRSDGIs are related at least in part due to socioeconomic measures like poverty and health expenditures.
- These results further confirm social determinants of health as major factors in health inequalities in the SDG era.

**What do the new findings imply?**

- There is an urgent need to focus on closing the gaps related to social determinants of health; in countries like Mexico, where social inequalities are widespread, addressing health inequities could be a leverage towards equity.

## Introduction

The sustainable development goals (SDGs) agenda sets out a series of goals and accompanying targets and indicators to reach by 2030. Since the SDGs were adopted in September 2015, a number of global initiatives have launched measurement efforts to determine levels and progress in achieving the health-related SDGs.^1^ At present, 232 individual SDGs indicators are included in the global SDGs indicator framework.^1^ Of the original global SDGs framework, 12 goals, 33 targets and 57 indicators have been identified as health-related.^2^

As part of the Global Burden of Diseases, Injuries, and Risk Factors (GBD) Study 2015, a baseline assessment for 33 health-related SDG indicators (HRSDGIs) was generated, producing an overall summary indicator called the health-related SDGs index.^3^ Previous measures indicate that country-level performance for the health-related SDGs index varied greatly in 2016, demonstrating health inequalities by countries and levels of socioeconomic development.^4^ The GBD study uses highly standardised analytical approaches to produce comprehensive and comparable estimates across countries and over time. In the same way, it has developed robust methodological approaches to estimate subnational levels and trends, allowing for a deeper understanding of particular countries’ performance. In a previous analysis, Mexico scored 67 on the health-related SDGs index (the highest score was for Singapore, at 86.8, and the lowest was for Afghanistan, at 10.9).^4^ Such performance rankings potentially mask substantial differences in subnational performance, of which are likely directly related to broader socioeconomic inequalities and local development challenges that threaten future progress toward the SDGs.

Mexico has sought to improve development and its performance on various social indicators in recent years. For instance, according to Mexico’s multidimensional poverty metric,^5^ the proportion of people living in extreme poverty decreased from 11.0% in 2008 to 7.4% in 2018—or 3.7 million fewer individuals living in such conditions. By 2018, 83.8% of Mexican reported access to prepaid health services, an increase from 62.0% in 2008.^5 6^ Amid such progress, however, 20.2 million Mexicans still lack access to health services, emphasising persisting inequalities in access to and coverage of care. In addition, increasingly more Mexicans are turning to private services with high out-of-pocket expenditures,^7 8^ emphasising challenges in both the access and perception of quality care found in public services.^7 8^ Such inequalities are related to many socioeconomic factors, including but not limited to sex, age, ethnicity, income and education; further, these inequalities are directly related to considerable variations in health-related outcomes and SDGs attainment levels at the subnational level, pointing to equally sizeable challenges to overcome.^9 10^

Previous analyses have examined health inequalities in Mexico, in particular related to effective coverage of health services and how such gaps in effective coverage were related to socioeconomic status and health outcomes (eg, populations with higher socioeconomic status had better health service coverage and reported better outcomes^11–13^). These analyses also found a 20% difference in overall effective coverage among Mexican states with the lowest and highest performance.^14^ Addressing such health inequities is a key development challenge,^15^ and lacking strong, timely evidence on their trends make their redress even more challenging. Primary obstacles include information gaps on the barriers and facilitating factors that affect healthcare access among populations facing inequalities; and inadequate knowledge about the strategies, interventions, tools and instruments available for measures based on equity.^16^

In this study, we provide an in-depth analysis of the HRSDGIs in Mexico from 1990 to 2017, aiming to quantify gaps that can be attributed to socioeconomic differences and inequalities between Mexican states. Understanding health inequalities and development gaps is an important input for decision-makers, allowing the establishment of priorities, policy development and implementation to improve development and SDG performance in the future.

Mexico, like other low-income to middle-income countries, is a diverse and socially heterogeneous country with marked social inequalities that affect healthcare access and health outcomes. Although SDG commitments are meant to occur at the country level, it is unlikely that many HRSDGI targets will be achieved if disparities persist—particularly among southern states with historically lower performance on various health metrics.

## Methods

### Overview of GBD and SDGs health-related indicators

GBD provides age-specific, sex-specific and location-specific estimates (including subnational-level for select countries) of all-cause and cause-specific mortality and morbidity, risk factor exposure and mortality and morbidity attributable to these risks, from 1990 to the most recent year for which data are available.^17–19^ Further details on GBD 2017, which covers 1990–2017, are available elsewhere.^19–22^

The entire GBD time series is updated in full for each research cycle with improved methods and data sources. GBD uses highly standardised and validated approaches applied to all available data sources, adjusted for major sources of bias.^3 4 19^ As with GBD 2017, this analysis complies with the Guidelines for Accurate and Transparent Health Estimates Reporting recommendations.

GBD draws from country-generated data that then synthesised alongside other data sources when country-specific data on specific indicators are not available. In the case of Mexico, 142 different sources informed estimates for the HRSDGIs in GBD 2017. Most of these data were derived from national health surveys, population censuses and surveys by the National Institute of Statistics and Geography (Inegi, by its acronym in Spanish), as well as other topic-specific surveys in Mexico. One particular strength for this analysis is the vast amount of data available from Mexico’s health information systems, health and general surveys, and the strong statistics system in Mexico.

We use GBD 2017 estimates for this analysis, which included 41 HRSDGIs, and of those, 40 were included in the health-related SDGs index.^19^ For this analysis, we used the unscaled values (ie, underlying estimates) for each HRSDGI reported by GBD 2017 for the years 1990, 2000, 2010 and 2017; more in-depth information on HRSDGI definitions and their measurement are reported elsewhere,^14^ as is the method for calculating the overall index.^3^

We also included a measure of financial protection—the percentage of population without health insurance—as reported by the Mexico’s National Council on Social Policy Evaluation for 2008, 2012, 2018; this indicator has not been previously reported regarding the HRSDGIs for Mexico.^5^

### Socioeconomic ranking variable

We ranked states using the Socio-demographic Index (SDI) included from GBD and the marginalisation index (MI) scores produced by the National Population Council (Conapo by its acronym in Spanish). SDI a composite indicator of development that includes the total fertility rate of women under the age of 25 years old; mean years of education among those 15 or more years old; and lag distributed income per capita.^23^ In turn, the MI is a multidimensional indicator that measures the intensity of deprivation based on nine forms of exclusion grouped into four dimensions: education, housing, population distribution and monetary income.^24^ At the state level, the SDI and the MI had a correlation coefficient of −0.97 in 1990 and −0.93 in 2017, so are equivalent for the analysis. We report on both, the MI is widely used in Mexico and the SDI provides a global comparison.

### Inequalities

For this analysis, we estimated the concentration index (CI) for 43 indicators: the 40 HRSDGIs reported for GBD 2017 and by cadre for human resources for health (physicians, nurses and pharmacists) using SDI and MI as stratifiers. We also examined the effects of basing this stratification on the percentage of individuals living in poverty.^5^

### Concentration index

The CI is a summary measure of inequality. CI measures the degree of socioeconomic-related inequality for a specific health indicator. The value obtained indicates how distributed the indicator is among the population ordered by a socioeconomic variable. For that purpose, we used both SDI and MI. CI is derived from a concentration curve for each indicator by states ordered by their SDI or MI. The CI is then defined as two times the area between the concentration curve and the line of equality (the 45-degree line), and ranges from −1 to 1, wherein 0 represents an absence of inequality. In this analysis, negative values indicate pro-poor indicators, or that states with the lowest SDI concentrate around higher values of the indicator^25 26^ and the opposite pattern for MI. The CI is estimated by:

$$CI=\frac{2}{N^{2}\times \mu_{h}}\times \sum_{i}^{n}h_{i}\times r_{i}$$

where $h_{i}$ is the health indicator at the state i, *μ*_*h*_ is the mean of the health indicator and $r_{i}$ is the fractional rank of state for SDI or MI, with the N state as the highest index value (ie, the most developed). As has been discussed by Wagstaff,^26^ for health indicators that are bounded—such as those with prevalence included in this analysis—standard CI estimates are not bound between −1 and 1. To address this, a normalisation process occurred, dividing the CI by 1 minus the mean:

$$CI=\frac{\frac{2}{N^{2}\times \mu_{h}}\times \sum_{i}^{n}h_{i}\times r_{i}}{1-\mu_{h}}$$

We estimated the standard CI for indicators reported as rate or incidence, and the Wagstaff-adjusted CI for those reported as percentage or prevalence. Data processing was conducted using Stata V.15.^27^ We tested the changes between CI in 1990 and 2017 for each indicator using a t-test with 31 degrees of freedom (32 states minus 1).

### Decomposition analysis

To identify factors that could explain health inequalities, we implemented a decomposition analysis for the estimated CI when it was statistically different from zero for the year 2017. In particular, we followed the Wagstaff approach, assuming an additive model where inequalities in a set of variables are used in a regression model as explanatory variables for the inequality in health. In particular, we aimed to discern the weight on health inequality of the allocation of resources at the state level, measured as both the public expenditure on health per capita and the public expenditure on health as a percentage of the state-level gross domestic product (GDP).

Dependent variables for the CI are the health indicators described earlier. The five independent variables are state-level data on GDP, health expenditures per capita, percentage of GDP spent on health, illiteracy index and poverty index.^5 24 28^ We can interpret the estimated coefficients as the statistical association between the independent variable and health indicator, not a causal relationship. This is important because socioeconomic variables likely have bidirectional relationships for health and health inequalities (eg, better health may have an independent causal effect on literacy). We used the following model to estimate CI:

$$CI=\sum k\left(\beta_{k}{\overset{-}{X}}_{k}/\mu \right)CI_{k}+GCI_{\varepsilon }/\mu$$

where μ is the mean of the health indicator, ${\overset{-}{X}}_{k}$ is the mean of $X_{k}$, $CI_{k}$ is the concentration index of $X_{k}$, and $GCI_{\varepsilon }$ is the generalised concentration index of the error term.^29 30^

### Patient and public involvement

All data used for these analyses are drawn from GBD 2017 study estimates. For these specific analyses, there were no public nor patient involvement.

## Results

**Figure 1:**
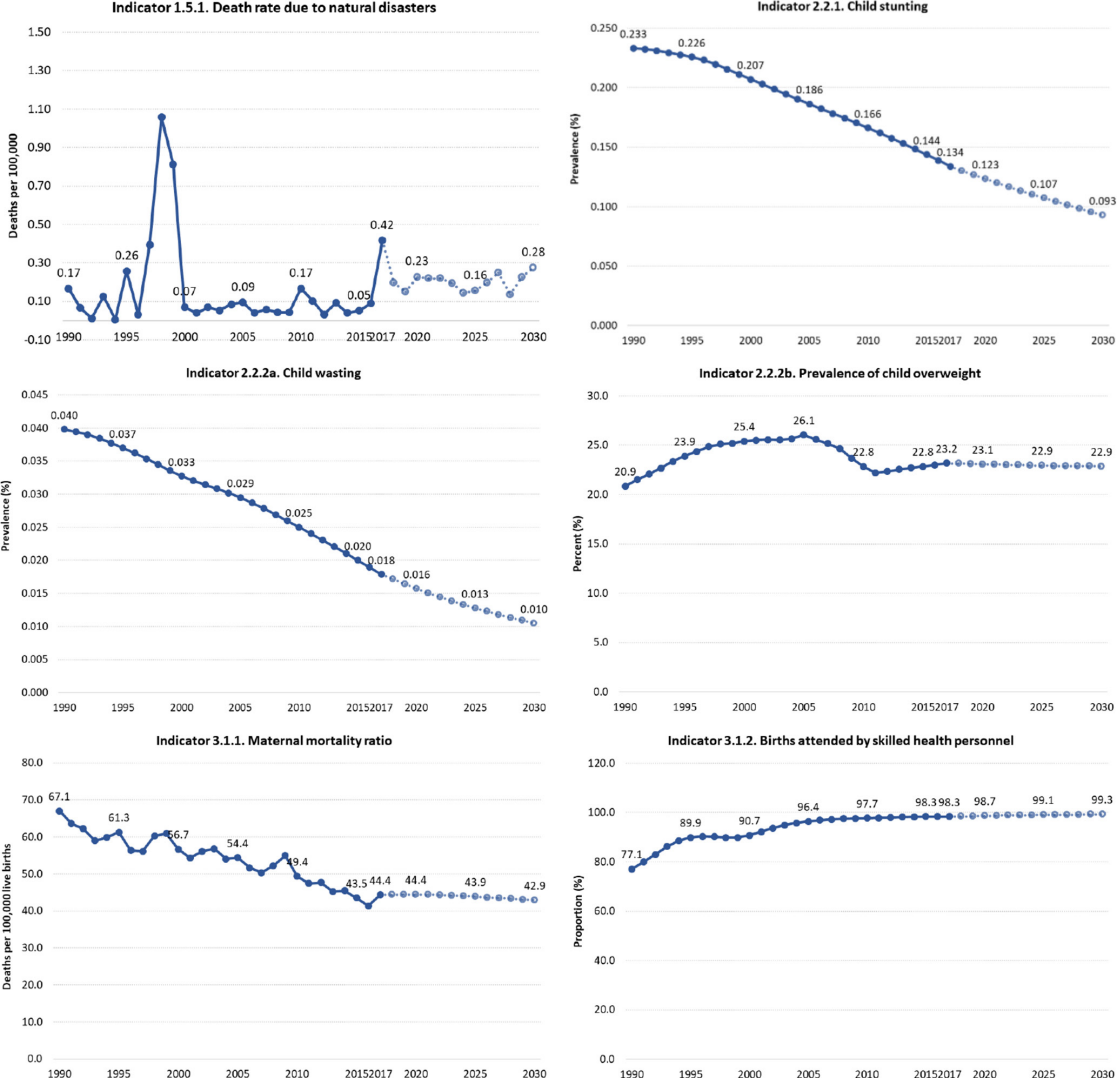

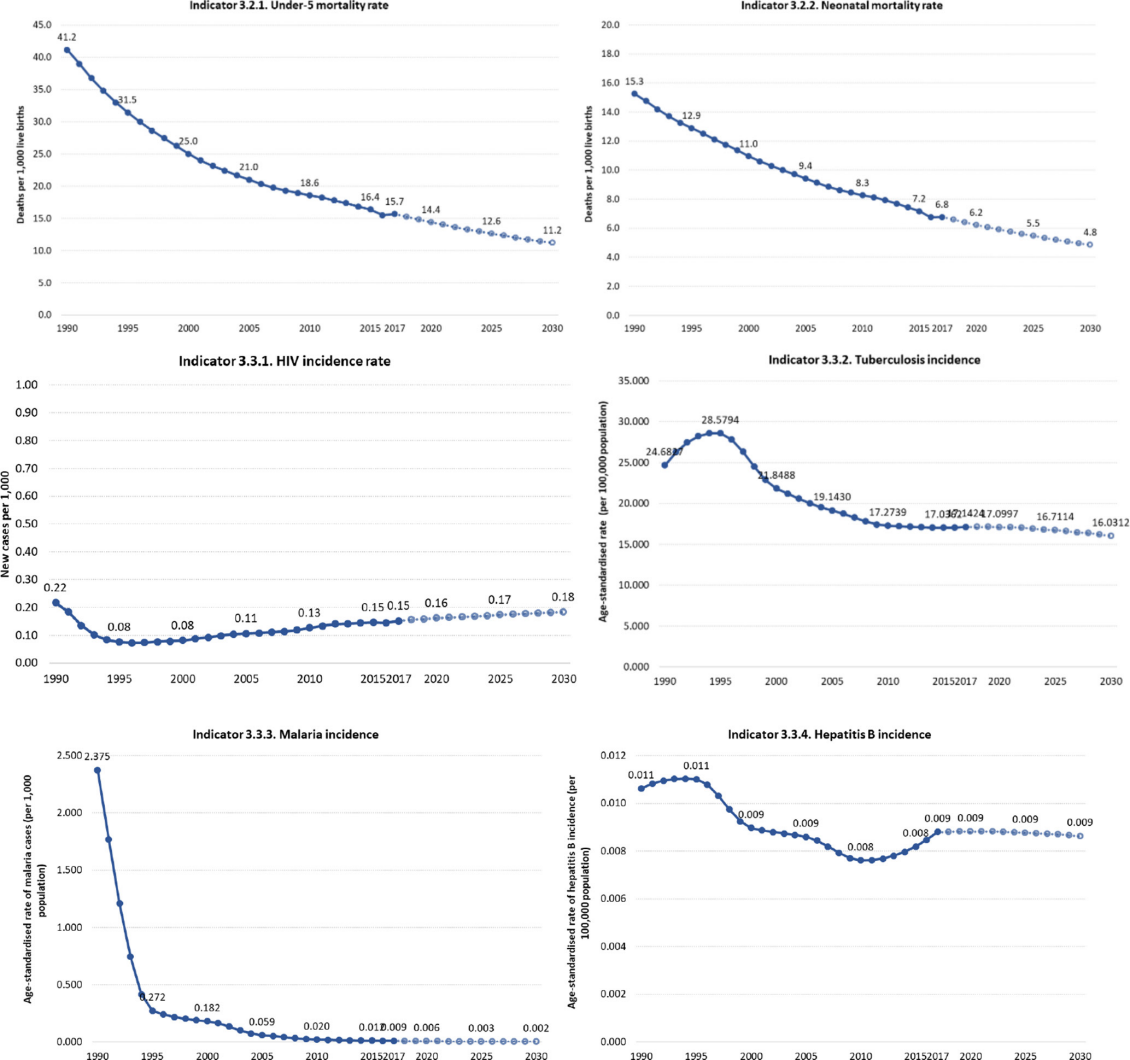

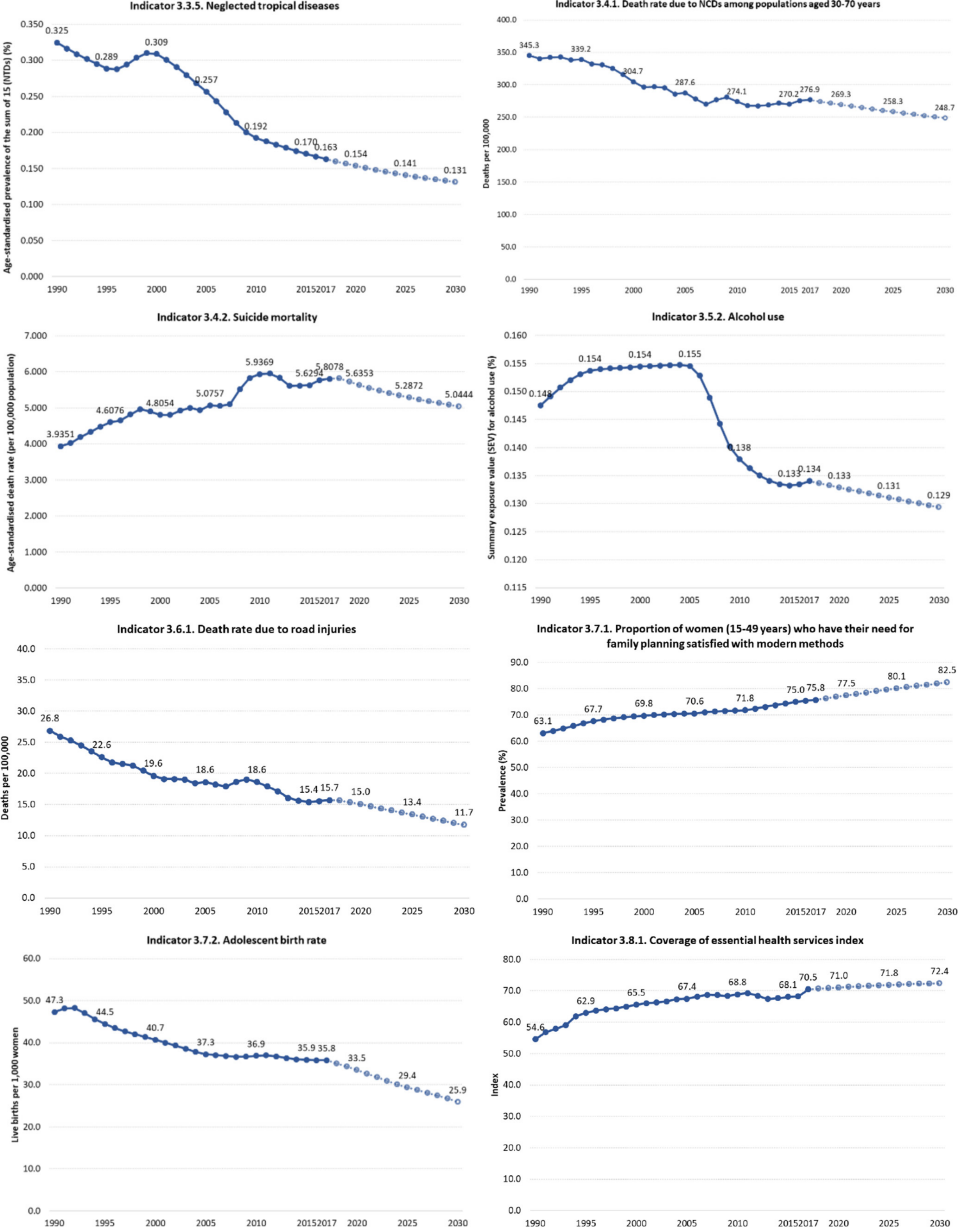

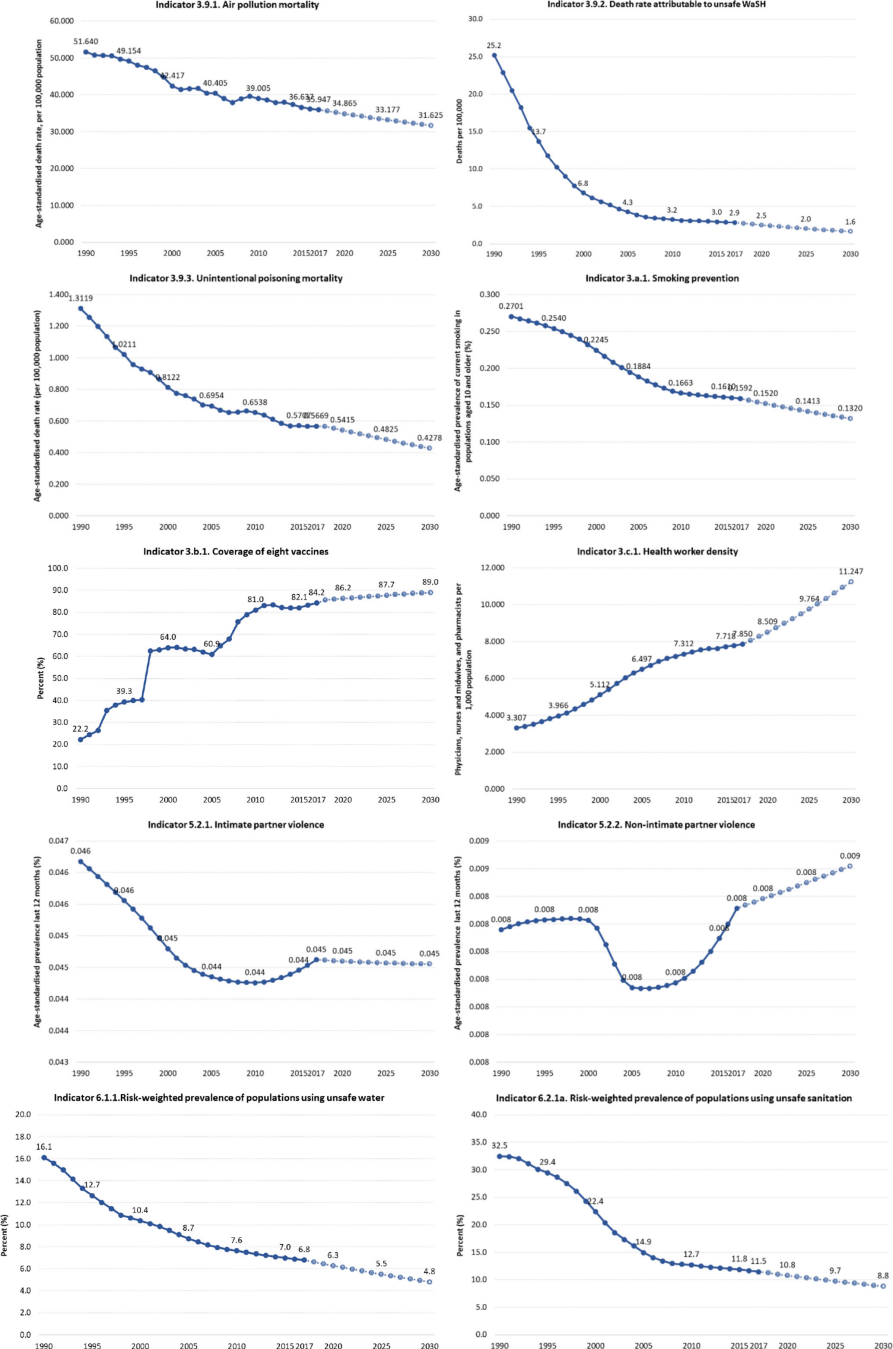

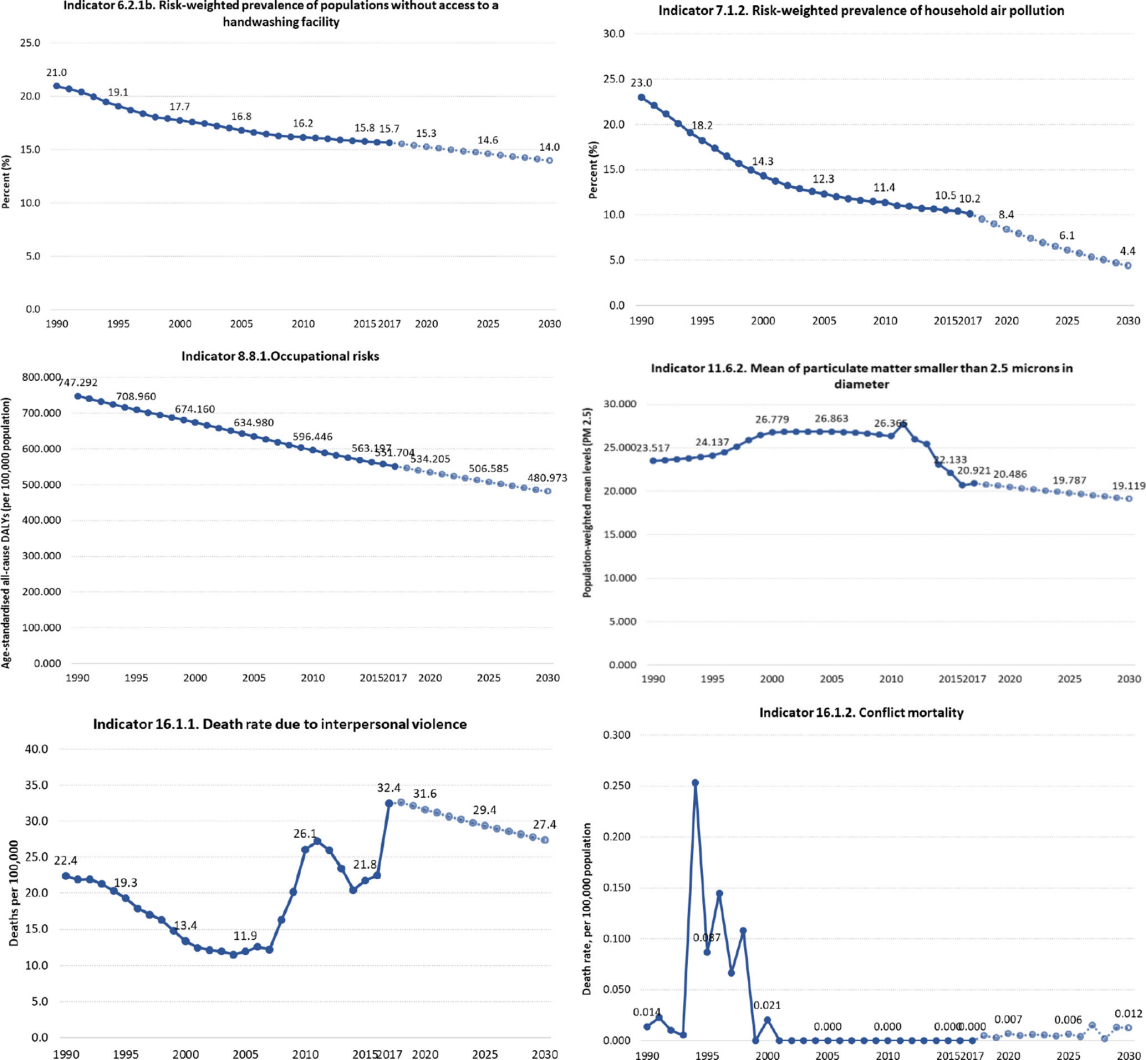

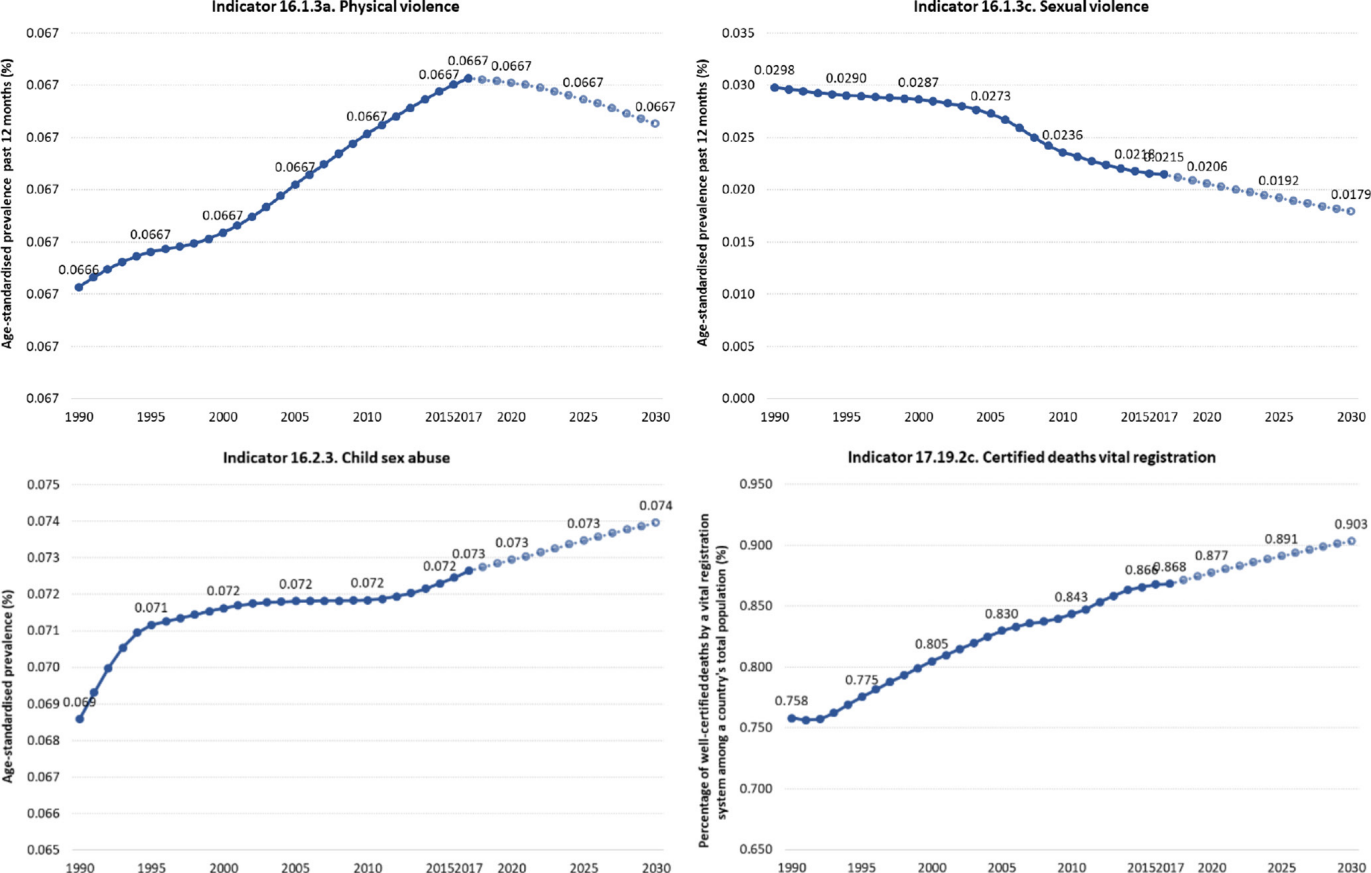
Trends in health-related sustainable development goals indicator in Mexico, 1990–2017.

In 2017, Mexico’s health-related index was 64.2 (uncertainty interval (UI) 62.9–65.4). This value represents an annualised rate of change (ARC) of 1.9% from the index value in 1990 of 46.3 (UI 45.0–47.4). Nevertheless, projected performance indicates that by 2030, the country will reach a value of 70.6 (UI 66.3–73.2) with an ARC of 0.7% from 2017 to 2030 (figure 1).

Figure 1 shows national HRSDGI trends from 1990 to 2030. Overall, most indicators are improving, but for some indicators, trends show deteriorating performance: prevalence of child overweight, child sex abuse, conflict mortality, incidence of hepatitis B, incidence of HIV, homicides, suicide, prevalence of population without access to a handwashing facility, intimate partner violence and maternal mortality.

**Figure 2:**
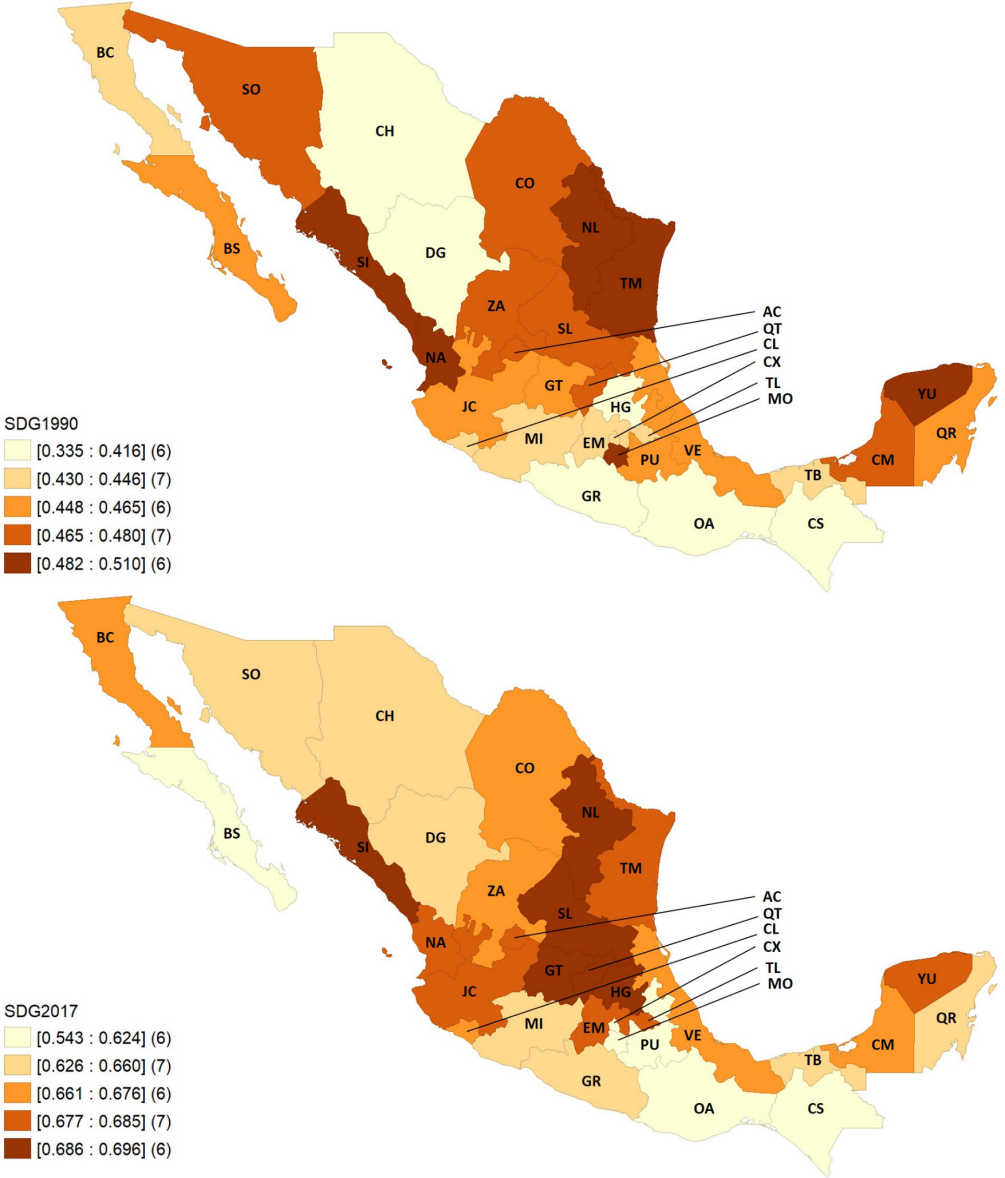
SDG index by state in 1990 and 2017, Mexico. AC, Aguascalientes; BC, Baja California; BS, Baja California Sur; CM, Campeche; CS, Chiapas; CH, Chihuahua; CO, Coahuila: CL, Colima; CX, Ciudad de México; DG, Durango; EM, Estado de México; GT, Guanajuato; GR, Guerrero; HG, Hidalgo; JC, Jalisco; MI, Michoacán; MO, Morelos; NA, Nayarit; NL, Nuevo León; OA, Oaxaca; PU, Puebla; QT, Querétaro; QR, Quintana Roo, SDG, sustainable development goal; SL, San Luis Potosí; SI, Sinaloa; SO, Sonora; TB, Tabasco; TM, Tamaulipas; TL, Tlaxcala; VE, Veracruz; YU, Yucatán; ZA, Zacatecas.

The health-related SDGs index is relative was to the performance of 195 countries and territories over time; as such, Mexico shows notable progress among country rankings, moving from fifth decile in 1990 to the fourth in 2017, and to the third decile in the projection to 2030. Within Mexico, substantial variation emerged on overall index performance (figure 2). In 1990, the highest index value was for Nuevo León, 51.0 (UI 47.2–53.8), and the lowest was in Chihuahua, 33.5 (UI 30.0–36.0). In 2017, the highest score was still in Nuevo León, 69.6 (UI 66.3–72.1), while the lowest was in Puebla, 54.2 (UI 52.5–55.9). The pace of progress also varied by state, with Morelos (0.5%) showing the lowest ARC from 1990 to 2017 and Chihuahua having the highest (2.3%). The above summarises uneven progress among states, with a tendency to concentrate the summary index.

Across indicators (heatmaps in the section 1 in online supplemental appendix), states varied widely between each other and then across indicators within each state for 1990 and 2017. Overall, southern states including Chiapas, Oaxaca, Puebla and Guerrero had among the worst performances across HRSDGIs. Of the 40 analysed indicators, the trend for 1990–2017 is improving for 22 HRSDGIs, is remaining relatively constant for 8 indicators, and 10 are worsening (section 2 in online supplemental appendix). At the national level, based on past trends, Mexico is only likely to meet the SDG targets for six HRSDGIs (out of the 25 with defined targets): maternal mortality ratio, skilled birth attendance (SBA), under-5 mortality, neonatal mortality, malaria incidence and well-certified death registry.

10.1136/bmjgh-2020-002382.supp1 Supplementary data

### Inequalities across states

CI estimates for the HRSDGIs and the additional health worker cadres are reported for 1990, 2000, 2010 and 2017 in table 1. Of the 43 indicators, evidence of inequality was identified for 30 (ie, CI values were larger than the standard threshold of (0.2)). Of these 30 indicators, inequalities affecting lower development states were found for 23 indicators (ie, positive values for CI) and inequalities affecting higher development states were identified for 7 indicators (ie, negative values for CI).

**Table 1:** Concentration index (SE) for health-related sustainable development goals indicator in Mexico using both SDI and marginalisation index, 1990, 2000, 2010 and 2017

No.	Indicator	SDI					Margnalisation index					Indicator description
		1990	2000	2010	2017	t value	1990	2000	2010	2017	t value*	
1	Adol Birth Rate	−0.048***	−0.057***	−0.041***	−0.043***	0.368	0.047***	0.052***	0.037***	0.039***	−0.588	Indicator 3.7.2: number of live births per 1000 women aged 10–19 years old
		(0.011)	(0.011)	(0.009)	(0.008)		(0.011)	(0.012)	−0.01	(0.008)		
		0.000	0.000	0.000	0.000		0.000	0.000	0	0.000		
2	Air Poll Mort	0.004	0.027*	0.016	0.008	0.226	−0.005	−0.032**	−0.015	−0.009	−0.226	Indicator 3.9.1: age-standardised death rate attributable to household air pollution and ambient air pollution (per 100 000 population)
		(0.012)	(0.013)	(0.010)	(0.013)		(0.012)	(0.013)	(0.010)	(0.013)		
		0.777	0.051	0.136	0.543		0.668	0.017	0.148	0.491		
3	Alcohol Use	0.048***	0.049***	0.046***	0.047***	−0.118	−0.048***	−0.050***	−0.044***	−0.045***	0.354	Indicator 3.5.2: risk-weighted prevalence of alcohol consumption, as measured by the summary exposure value (SEV) for alcohol use (%)
		(0.006)	(0.006)	(0.005)	(0.006)		(0.006)	(0.005)	(0.005)	(0.006)		
		0.000	0.000	0.000	0.000		0.000	0.000	0.000	0.000		
11	FP Need Met, Mod	0.105***	0.102***	0.102***	0.091***	−0.482	−0.097***	−0.094***	−0.085***	−0.077***	0.642	Indicator 3.7.1: proportion of women of reproductive age (15–49 years) who have their need for family planning satisfied with modern methods (%)
		(0.022)	(0.022)	(0.019)	(0.019)		(0.023)	(0.023)	(0.022)	(0.021)		
		0.000	0.000	0.000	0.000		0.000	0.000	0.000	0.001		
13	HIV Incid	0.112***	−0.055	−0.036	−0.032	−2.671	−0.110***	0.072	0.068	0.057	3.142	Indicator 3.3.1: age-standardised rate of new HIV infections (per 1000 population)
		(0.035)	(0.043)	(0.042)	(0.041)		(0.035)	(0.043)	(0.041)	(0.040)		
		0.003	0.213	0.401	0.434		0.003	0.103	0.105	0.163		
14	Health Worker Dens	0.144***	0.139***	0.124***	0.111***	−2.917	−0.143***	−0.138***	−0.120***	−0.108***	2.750	Indicator 3.c.1: physicians, nurses and midwives, and pharmacists per 1000 population
		(0.008)	(0.010)	(0.008)	(0.008)		(0.009)	(0.010)	(0.010)	(0.009)		
		0.000	0.000	0.000	0.000		0.000	0.000	0.000	0.000		
15	Health Worker Dens Nurses Midwives	0.139***	0.133***	0.117***	0.106***	−2.453	−0.136***	−0.130***	−0.114***	−0.104***	2.153	Indicator 3.c.1b: nurses and midwives per 1000 population
		(0.010)	(0.011)	(0.009)	(0.009)		(0.011)	(0.012)	(0.011)	(0.010)		
		0.000	0.000	0.000	0.000		0.000	0.000	0.000	0.000		
16	Health Worker Dens Pharmacists	0.176***	0.162***	0.143***	0.125***	−0.890	−0.176***	−0.154***	−0.129***	−0.113**	1.085	Indicator 3.c.1c: pharmacists per 1000 population
		(0.039)	(0.041)	(0.041)	(0.042)		(0.039)	(0.042)	(0.043)	(0.043)		
		0.000	0.000	0.002	0.006		0.000	0.001	0.005	0.013		
17	Health Worker Dens Physicians	0.153***	0.150***	0.136***	0.121***	*−1.371*	−0.155***	−0.152***	−0.133***	−0.118***	1.585	Indicator 3 .c.1a: physicians per 1000 population
		(0.017)	(0.020)	(0.019)	(0.016)		(0.016)	(0.020)	(0.019)	(0.017)		
		0.000	0.000	0.000	0.000		0.000	0.000	0.000	0.000		
18	Hep B Incid	−0.015	−0.024	−0.038**	−0.045***	−1.229	0.013	0.026	0.035**	0.044***	1.270	Indicator 3.3.4: age-standardised rate of hepatitis B incidence (per 100 000 population)
		(0.020)	(0.018)	(0.014)	(0.014)		(0.020)	(0.018)	(0.014)	(0.014)		
		0.445	0.188	0.011	0.003		0.511	0.151	0.022	0.004		
22	Malaria Incid	−0.494***	−0.591**	−0.633**	−0.652*	−0.436	0.500***	0.543**	0.635**	0.651*	0.417	Indicator 3.3.3: age-standardised rate of malaria cases (per 1000 population)
		(0.158)	(0.234)	(0.254)	(0.326)		(0.158)	(0.238)	(0.254)	(0.326)		
		0.004	0.017	0.019	0.055		0.003	0.029	0.018	0.055		
23	Mat Mort Ratio	−0.122***	−0.075***	−0.066***	−0.064***	1.580	0.123***	0.077***	0.068***	0.062***	−1.639	Indicator 3.1.1: maternal mortality ratio (maternal deaths per 100 000 live births) in women aged 10–54 years
		(0.032)	(0.026)	(0.020)	(0.018)		(0.032)	(0.026)	(0.019)	(0.019)		
		0.001	0.007	0.002	0.002		0.001	0.006	0.001	0.003		
25	NCD Mort	0.075***	0.053***	0.039***	0.029***	−3.419	−0.074***	−0.060***	−0.035***	−0.030***	3.270	Indicator 3.4.1: age-standardised death rate due to cardiovascular disease, cancer, diabetes and chronic respiratory disease in populations aged 30–70 (per 100 000 population)
		(0.009)	(0.012)	(0.010)	(0.010)		(0.009)	(0.010)	(0.011)	(0.010)		
		0.000	0.000	0.001	0.007		0.000	0.000	0.003	0.004		
26	NTD Prev	−0.144***	−0.169***	−0.143***	−0.130***	0.728	0.142***	0.165***	0.137***	0.124***	−0.838	Indicator 3.3.5: age-standardised prevalence of the sum of 15 neglected tropical diseases (NTDs) (%)
		(0.009)	(0.014)	(0.018)	(0.017)		(0.010)	(0.015)	−0.019	(0.019)		
		0.000	0.000	0.000	0.000		0.000	0.000	0.000	0.000		
27	Neonatal Mort	−0.003	0.004	0.009	0.011	0.507	−0.003	−0.010	−0.011	−0.016	−0.471	Indicator 3.2.2: neonatal mortality rate (probability of dying during the first 28 days of life per 1000 live births)
		(0.020)	(0.016)	(0.017)	(0.019)		(0.020)	(0.016)	(0.017)	(0.019)		
		0.881	0.821	0.615	0.577		0.891	0.560	0.538	0.384		
31	Poisoning Mort	−0.048	−0.076*	−0.041	−0.045	0.055	0.039	0.033	0.054	0.035	−0.072	Indicator 3.9.3: age-standardised death rate due to unintentional poisonings (per 100 000 population)
		(0.039)	(0.044)	(0.045)	(0.038)		(0.040)	(0.046)	(0.044)	(0.039)		
		0.228	0.095	0.364	0.253		0.336	0.479	0.234	0.378		
32	Road Inj Mort	0.011	0.002	−0.009	−0.008	−0.726	−0.013	−0.011	0.013	0.008	0.802	Indicator 3.6.1: age-standardised death rate due to road injuries (per 100 000 population)
		(0.018)	(0.019)	(0.020)	(0.019)		(0.018)	(0.019)	(0.020)	(0.019)		
		0.553	0.911	0.648	0.661		0.469	0.558	0.528	0.663		
35	Skilled Birth Attend	0.288***	0.340***	0.230	0.263**	−0.175	−0.297***	−0.251**	−0.292**	−0.274**	0.162	Indicator 3.1.2: proportion of births attended by skilled health personnel (%)
		(0.087)	(0.108)	(0.139)	(0.113)		(0.086)	(0.116)	(0.135)	(0.113)		
		0.002	0.004	0.110	0.027		0.002	0.039	0.039	0.021		
36	Smoking Prev	0.142***	0.143***	0.119***	0.110***	−1.151	−0.147***	−0.156***	−0.131***	−0.122***	0.976	Indicator 3.a.1: age-standardised prevalence of current smoking in populations aged 10 and older (%)
		(0.017)	(0.020)	(0.024)	(0.022)		(0.016)	(0.016)	(0.022)	(0.020)		
		0.000	0.000	0.000	0.000		0.000	0.000	0.000	0.000		
37	Suicide Mort	0.042	0.031	0.029	0.032	−0.231	−0.033	−0.029	−0.014	−0.027	0.139	Indicator 3.4.2: age-standardised death rate due to self-harm (per 100 000 population)
		(0.033)	(0.034)	(0.029)	(0.028)		(0.033)	(0.034)	(0.029)	(0.028)		
		0.209	0.367	0.328	0.266		0.327	0.394	0.644	0.349		
38	TB Incid	−0.066	−0.058	−0.030	−0.046	0.293	0.078*	0.066	0.075	0.070	−0.119	Indicator 3.3.2: age-standardised rate of tuberculosis cases (per 100 000 population)
		(0.043)	(0.042)	(0.061)	(0.053)		(0.043)	(0.041)	(0.059)	(0.052)		
		0.137	0.174	0.630	0.388		0.077	0.119	0.217	0.184		
39	UHC Serv Cov Index	0.050***	0.048***	0.039***	0.042***	−0.595	−0.048***	−0.041***	−0.034***	−0.036***	0.807	Indicator 3.8.1: coverage of essential health services, as defined by the UHC service coverage index comprised the coverage of nine tracer interventions and 32 causes amenable to healthcare (scale of 0–100)
		(0.010)	(0.009)	(0.008)	(0.009)		(0.011)	(0.010)	(0.009)	(0.010)		
		0.000	0.000	0.000	0.000		0.000	0.000	0.001	0.001		
40	Under-5 Mort	−0.105***	−0.095***	−0.084***	−0.075***	1.500	0.101***	0.086***	0.078***	0.066***	−1.635	Indicator 3.2.1: under-5 mortality rate (probability of dying before the age of 5 per 1000 live births)
		(0.012)	(0.012)	(0.017)	(0.016)		(0.013)	(0.014)	(0.018)	(0.017)		
		0.000	0.000	0.000	0.000		0.000	0.000	0.000	0.001		
41	Vaccine Cov	−0.065***	−0.072***	−0.134***	−0.148***	−1.928	0.066***	0.076***	0.142***	0.153***	2.062	Indicator 3.b.1: coverage of eight vaccines in target populations (%)
		(0.022)	(0.020)	(0.034)	(0.037)		(0.022)	(0.019)	(0.033)	(0.036)		
		0.006	0.001	0.000	0.000		0.004	0.000	0.000	0.000		
42	WaSH Mort	−0.327***	−0.304***	−0.235***	−0.211***	*1.647*	0.334***	0.299***	0.227***	0.205***	−1.855	Indicator 3.9.2: age-standardised death rate attributable to unsafe water, sanitation and hygiene (WaSH) (per 100 000 population)
		(0.055)	(0.048)	(0.047)	(0.044)		(0.053)	(0.049)	(0.048)	(0.045)		
		0.000	0.000	0.000	0.000		0.000	0.000	0.000	0.000		
21	Int Partner Viol	−0.055	−0.063	−0.043	−0.076	−0.198	0.045	0.043	0.035	0.042	−0.028	Indicator 5.2.1: age-standardised prevalence of ever-partnered women aged 15 years and older who experienced physical or sexual violence by a current or former intimate partner in the last 12 months (%)
		(0.073)	(0.075)	(0.080)	(0.077)		(0.073)	(0.076)	(0.081)	(0.078)		
		0.453	0.408	0.597	0.335		0.541	0.573	0.665	0.597		
28	Non-Int Partner Sex Viol	−0.101	−0.095	−0.073	−0.078	0.256	0.097	0.102	0.098	0.102	0.056	Indicator 5.2.2: age-standardised prevalence of women aged 15 years and older who experienced physical or sexual violence by non-intimate partner in the last 12 months (%)
		(0.064)	(0.064)	(0.061)	(0.063)		(0.064)	(0.063)	(0.060)	(0.062)		
		0.122	0.147	0.245	0.228		0.138	0.117	0.115	0.113		
20	Hygiene	−0.251***	−0.178***	−0.076***	−0.061***	7.121	0.261***	0.186***	0.079***	0.062***	−8.372	Indicator 6.2.1b: risk-weighted prevalence of populations without access to a handwashing facility, as measured by the SEV for unsafe hygiene (%)
		(0.026)	(0.022)	(0.009)	(0.006)		(0.023)	(0.019)	(0.008)	(0.006)		
		0.000	0.000	0.000	0.000		0.000	0.000	0.000	0.000		
33	Sanitation	−0.213***	−0.267***	−0.296***	−0.267***	−0.682	0.218***	0.256***	0.301***	0.272***	0.694	Indicator 6.2.1a: risk-weighted prevalence of populations using unsafe or unimproved sanitation, as measured by the SEV for unsafe sanitation (%)
		(0.070)	(0.047)	(0.041)	(0.037)		(0.069)	(0.049)	(0.039)	(0.036)		
		0.005	0.000	0.000	0.000		0.004	0.000	0.000	0.000		
43	Water	−0.397***	−0.381***	−0.281***	−0.244***	3.201	0.410***	0.398***	0.295***	0.255***	−3.744	Indicator 6.1.1: risk-weighted prevalence of populations using unsafe or unimproved water sources, as measured by the SEV for unsafe water (%)
		(0.038)	(0.045)	(0.034)	(0.029)		(0.033)	(0.040)	(0.029)	(0.025)		
		0.000	0.000	0.000	0.000		0.000	0.000	0.000	0.000		
12	HH Air Poll	−0.427***	−0.475***	−0.488***	−0.463***	−0.856	0.429***	0.480***	0.498***	0.473***	1.198	Indicator 7.1.2: risk-weighted prevalence of household air pollution, as measured by the SEV for household air pollution (%)
		(0.020)	(0.030)	(0.038)	(0.037)		(0.018)	(0.026)	(0.033)	(0.032)		
		0.000	0.000	0.000	0.000		0.000	0.000	0.000	0.000		
29	Occ Burden	−0.027***	−0.021***	−0.017***	−0.014**	*1.664*	0.025***	0.017**	0.015**	0.011*	*−1.650*	Indicator 8.8.1: age-standardised all-cause disability-adjusted life year rates attributable to occupational risks (per 100 000 population)
		(0.006)	(0.006)	(0.006)	(0.005)		(0.006)	(0.007)	(0.006)	(0.006)		
		0.000	0.003	0.007	0.012		0.000	0.021	0.013	0.057		
24	Mean PM2.5	0.027*	0.041***	0.044***	0.047***	1.010	−0.025*	−0.037**	−0.039***	−0.040***	−0.758	Indicator 11.6.2: population-weighted mean levels of fine particulate matter smaller than 2.5 μm in diameter (PM2.5)
		(0.014)	(0.013)	(0.012)	(0.014)		(0.014)	(0.014)	(0.013)	(0.014)		
		0.064	0.005	0.001	0.002		0.096	0.012	0.005	0.010		
6	Child Sex Abuse	−0.046***	−0.034***	−0.030***	−0.031***	0.881	0.046***	0.034***	0.030***	0.031***	−0.881	Indicator 16.2.3: age-standardised prevalence of women and men aged 18–29 years who experienced sexual violence by age 18 (%)
		(0.013)	(0.010)	(0.009)	(0.011)		(0.013)	(0.010)	(0.009)	(0.011)		
		0.001	0.002	0.003	0.007		0.001	0.002	0.004	0.007		
9	Conflict Mort	0.071	0.028	0.000	0.000	−0.362	0.004	−0.017	0.000	0.000	−0.020	Indicator 16.1.2: death rate due to conflict and terrorism (per 100 000 population)
		(0.196)	(0.071)	(0.000)	(0.000)		(0.196)	(0.071)	(0.000)	(0.000)		
		0.720	0.698		.		0.983	0.809		.		
19	Homicide	−0.104**	−0.085*	0.081	−0.028	1.303	0.099*	0.068	−0.010	0.034	−1.099	Indicator 16.1.1: age-standardised death rate due to interpersonal violence (per 100 000 population)
		(0.050)	(0.048)	(0.124)	(0.030)		(0.051)	(0.049)	(0.125)	(0.030)		
		0.049	0.087	0.521	0.366		0.060	0.177	0.934	0.264		
30	Physical Violence	0.000	0.000***	0.000**	−0.000	0.000	−0.000	−0.000***	−0.000*	0.000	0.000	Indicator 16.1.3a: age-standardised prevalence of physical violence experienced by populations in the past 12 months (%)
		(0.000)	(0.000)	(0.000)	(0.000)		(0.000)	(0.000)	(0.000)	(0.000)		
		0.106	0.001	0.048	0.228		0.169	0.000	0.066	0.170		
34	Sexual Violence	0.030***	0.014***	0.007***	0.005**	−5.590	−0.031***	−0.013***	−0.008***	−0.005***	5.814	Indicator 16.1.3c: age-standardised prevalence of sexual violence experienced by populations in the past 12 months *(%)*
		(0.004)	(0.003)	(0.002)	(0.002)		(0.004)	(0.003)	(0.002)	(0.002)		
		0.000	0.000	0.003	0.018		0.000	0.000	0.000	0.007		
4	Cert Death Reg	0.158***	0.130***	0.069***	0.048***	−3.280	−0.157***	−0.122***	−0.071***	−0.048***	3.250	Indicator 17.19.2 c: percentage of well-certified deaths by a vital registration system among a country’s total population (%)
		(0.030)	(0.026)	(0.013)	(0.015)		(0.030)	(0.027)	(0.013)	(0.015)		
		0.000	0.000	0.000	0.003		0.000	0.000	0.000	0.002		
10	Disaster Mort	0.284	−0.083	−0.079	−0.295	−1.266	−0.355	0.087	0.021	0.268	1.366	Indicator 1.5.1: death rate due to exposure to forces of nature (per 100 000 population)
		(0.394)	(0.065)	(0.077)	(0.232)		(0.392)	(0.065)	(0.079)	(0.233)		
		0.476	0.212	0.313	0.215		0.372	0.187	0.788	0.260		
5	Child Overweight	0.148***	0.133***	0.086***	0.084***	−3.119	−0.142***	−0.126***	−0.082***	−0.077***	2.867	Indicator 2.2.2b: prevalence of overweight in children aged 2–4 (%)
		(0.015)	(0.015)	(0.014)	(0.014)		(0.017)	(0.017)	(0.015)	(0.015)		
		0.000	0.000	0.000	0.000		0.000	0.000	0.000	0.000		
7	Child Stunting	−0.118***	−0.118**	−0.111**	−0.137***	−0.340	0.115***	0.086*	0.105**	0.128***	0.227	Indicator 2.2.1: prevalence of stunting in children under 5 years (%)
		(0.041)	(0.043)	(0.043)	(0.038)		(0.042)	(0.046)	(0.044)	(0.039)		
		0.008	0.011	0.015	0.001		0.010	0.068	0.023	0.003		
8	Child Wasting	−0.070***	−0.042*	−0.034	−0.035*	1.207	0.071***	0.042*	0.025	0.028	*−1.520*	Indicator 2.2.2a: prevalence of wasting in children under 5 years (%)
		(0.021)	(0.022)	(0.021)	(0.020)		(0.020)	(0.022)	(0.022)	(0.020)		
		0.002	0.062	0.121	0.084		0.001	0.063	0.251	0.167		
			2008	2012	2018			2008	2012	2018		
45	Financial Protection		−0.187***	−0.061**	−0.010	4.306		0.163***	0.043	−0.006	−3.823	
			(0.031)	(0.027)	(0.027)			(0.035)	(0.028)	(0.027)		
			0.000	0.031	0.704			0.000	0.142	0.818		

*t value for the difference between 2017 and 1990; reference values for one-side tail and 30 freedom degrees: 1.6973 for p<0.05 and 1.3104 for p<0.10

NCD, non-communicable disease.

Focusing only SDG 3—the SDG goal expressly focused—18 out of 25 SDG3 indicators showed inequalities among Mexican states. Of note, CI values were close to 0, which suggests lower evidence of inequality, for the following HRSDGIs: deaths attributable to air pollution, HIV incidence, neonatal mortality rate, death rate for unintentional poisoning, death rate due to road injuries, death rate due to self-harm or Tabasco incidence.

Overall, our CI estimates suggest relatively low inequality for a subset of SDG3 (ie, below the 0.2 threshold is considered a signal of high inequality^31^). The main exceptions were SBA, malaria incidence, and the death rate attributable to unsafe water, sanitation and hygiene (WaSH). These particular HRSDGIs reflect various neglected health issues and vulnerabilities, which are unequally distributed and thus experienced throughout Mexico.

SBA was higher in more developed states, so CIs had positive values from 1990 to 2017. In 2017, the CI estimate was 0.263 (SE of 0.113, p=0.027), which was a 9% decrease from the CI estimate in 1990 (0.288 (SE 0.087, p=0.002)). For malaria, the inequality increased since 1990, with more negative values among less developed states: the CI for malaria incidence was −0.494 (SE of 0.158, p<0.004) in 1990 and increased to −0.652 (SE of 0.326, p=0.055) in 2017. For deaths attributable to unsafe (WaSH), CI values were negative for 1990–2017, indicating higher death rates among less developed states; however, the magnitude of inequality reflected by CI estimates decreased by 35% since 1990, from −0.327 (SE 0.055, p<0.001) in 1990 and −0.211 in 2017 (SE 0.044, p<0.001).

The two HRSDGIs under SDG 5 (gender equality)—prevalence of women experiencing intimate partner violence or the violence from a non-intimate partner—showed no evidence of inequality on the basis of CI estimates.

For SDG 6, which focuses on clean water and sanitation, all three HRSDGIs under this goal showed inequalities affecting populations living in less developed Mexican states. For the prevalence of populations without access to a handwashing facility, there was a clear trend of decreasing inequality over time: in 1990, the CI estimate was −0.251 (SE 0.026, p<0.001) and fell to −0.061 (SE 0.006, p<0.001) by 2017, a 76% decrease. In contrast, inequality in the prevalence in unsafe sanitation increased by 24%, from CI estimates of −0.213 (SE 0.070, p=0.005) in 1990 to −0.267 in 2017 (SE 0.037, p<0.001). For prevalence populations with unsafe water sources, inequality decreased from 1990 but absolute levels were still somewhat high. In 1990, the CI was −0.397 (SE 0.038, p=0.005), and then fell by 39% by 2017, to −0.244 (SE 0.029, p<0.001).

Under SDG 7, affordable and clean energy, the HRSDGI—prevalence of household air pollution—showed trends of increasing inequality for less developed states. In 1990, its CI was −0.427 (SE 0.020, p<0.001) in 1990 and then rose 8%, to −0.463 in 2017 (SE 0.037, p<0.001).

Under SDG 8, decent work and economic growth, the estimated CI for all-cause disability-adjusted life years attributable to occupational risked some evidence of decreasing inequality over time. SDG 11, the goal on sustainable cities and communities, had one HRSDGI: mean levels of fine particulate matter smaller than 2.5 μm in diameter (PM2.5). CI estimates showed higher values in more developed states, with inequality increasing.

Three HRSDGIs under SDG 16—the goal focused on peace, justice and strong institutions—showed no evidence of inequalities between less and more developed states: death rate due to conflict and terrorism, death rate due to interpersonal violence and prevalence of physical violence. In contrast, the HRSDGI on child sexual abuse (prevalence of men and women aged 18–29 years that experienced sexual violence by age 18) had a slightly higher concentration among less developed states, while the prevalence of sexual violence was slightly more concentrated among states with higher development.

For SDG 17, partnerships, the HRSDGI capturing the percentage of well-certified deaths showed some evidence of inequality such that worse values were found among less developed states. The death rate to natural disasters, a HRSDGI found in multiple SDG goals (including SDG 1, no poverty), did not have CI estimates that were statistically different from 0; subsequently, there was no clear evidence of inequalities among states for this indicator.

SDG 2, which calls for zero hunger, had three HRSDGIs indicating inequalities: prevalence of childhood stunting, wasting and overweight, with the two indicators related to undernourishment presenting inequality affecting less developed states, and the overweight being more concentrated among more developed states.

For the indicator on health insurance coverage, we found that in 2008 the was inequality among less developed states (CI of −0.187 (SE 0.031, p<0.01); by 2018, the concentration index (CI) estimate was not statistically distinguishable from zero, implying no evidence of inequality across states.

Details for all indicators analysed are reported in online supplemental appendix, section 3.

### Decomposition analysis

For the decomposition analysis (table 2), we included the 30 indicators that presented evidence of inequalities in the analysed years (ie, CI estimates were was statistically different from zero), and we ran the decomposition for 2017 on the following indicators: GDP per capita, public health expenditure per capita (PHE), percentage of GDP devoted to health (%HGDP), illiteracy rate and percentage of people living in poverty.

**Table 2:** Contribution to concentration index of health-related sustainable development goals indicator using gross domestic product (GDP) per capita by decomposition analysis in Mexico, 2017

Group	No.	Indicator		GDP pc	Health expenditures pc	GDP on health (%)	Illiteracy	Poverty	Indicator description
			CI	0.212	0.110	−0.124	−0.343	−0.193	
A	1	Adol Birth Rate	−0.043***	0.030	−0.022	−0.019	0.010	−0.036	Indicator 3.7.2: number of live births per 1000 women aged 10–19 years old
A	3	Alcohol Use	0.047***	0.000	0.012	−0.003	0.018	0.012	Indicator 3.5.2: risk-weighted prevalence of alcohol consumption, as measured by the summary exposure value (SEV) for alcohol use (%)
A	11	FP Need Met, Mod	0.091***	0.005	0.002	−0.008	0.013	0.010	Indicator 3.7.1: proportion of women of reproductive age (15–49 years) who have their need for family planning satisfied with modern methods (%)
A	14	Health Worker Dens	0.111***	0.006	0.025	−0.003	0.041	0.039	Indicator 3.c.1: physicians, nurses and midwives, and pharmacists per 1000 population
A	15	Health Worker Dens Nurses Midwives	0.106***	0.044	−0.014	−0.034	0.058	0.049	Indicator 3.c.1b: nurses and midwives per 1000 population
A	16	Health Worker Dens Pharmacists	0.125***	−0.239	0.182	0.208	−0.056	0.037	Indicator 3.c.1c: pharmacists per 1000 population
A	17	Health Worker Dens Physicians	0.121***	−0.063	0.100	0.053	0.009	0.130	Indicator 3.c.1a: physicians per 1000 population
A	18	Hep B Incid	−0.045***	0.020	−0.004	−0.005	0.035	−0.093	Indicator 3.3.4: age-standardised rate of hepatitis B incidence (per 100 000 population)
A	22	Malaria Incid	−0.652*	1.284	−0.742	−1.091	−0.657	0.485	Indicator 3.3.3: age-standardised rate of malaria cases (per 1000 population)
A	23	Mat Mort Ratio	−0.064***	0.039	0.015	−0.032	0.019	−0.098	Indicator 3.1.1: maternal mortality ratio (maternal deaths per 100 000 live births) in women aged 10–54 years
A	25	NCD Mort	0.029***	0.007	0.012	0.008	0.022	−0.024	Indicator 3.4.1: age-standardised death rate due to cardiovascular disease, cancer, diabetes and chronic respiratory disease in populations aged 30–70 (per 100 000 population)
A	26	NTD Prev	−0.130***	0.015	0.017	−0.005	−0.085	−0.050	Indicator 3.3.5: age-standardised prevalence of the sum of 15 neglected tropical diseases (NTDs) (%)
A	35	Skilled Birth Attend	0.263**	0.002	−0.001	−0.002	0.003	0.002	Indicator 3.1.2: proportion of births attended by skilled health personnel (%)
A	36	Smoking Prev	0.110***	−0.006	0.014	0.004	0.090	−0.007	Indicator 3.a.1: age-standardised prevalence of current smoking in populations aged 10 and older (%)
A	39	UHC Serv Cov Index	0.042***	−0.004	0.001	0.002	−0.005	0.019	Indicator 3.8.1: coverage of essential health services, as defined by the UHC service coverage index comprised the coverage of 9 tracer interventions and 32 causes amenable to healthcare (scale of 0–100)
A	40	Under-5 Mort	−0.075***	0.031	−0.008	−0.015	−0.004	−0.073	Indicator 3.2.1: under-5 mortality rate (probability of dying before the age of 5 per 1000 live births)
A	41	Vaccine Cov	−0.148***	0.004	−0.018	0.010	−0.010	−0.005	Indicator 3.b.1: coverage of eight vaccines in target populations (%)
A	42	WaSH Mort	−0.211***	0.182	−0.105	−0.171	−0.136	0.006	Indicator 3.9.2: age-standardised death rate attributable to unsafe water, sanitation and hygiene (WaSH) (per 100 000 population)
C	20	Hygiene	−0.061***	0.007	−0.007	−0.004	−0.038	−0.009	Indicator 6.2.1b: risk-weighted prevalence of populations without access to a handwashing facility, as measured by the SEV for unsafe hygiene (%)
C	33	Sanitation	−0.267***	0.063	−0.062	−0.098	−0.243	0.111	Indicator 6.2.1a: risk-weighted prevalence of populations using unsafe or unimproved sanitation, as measured by the SEV for unsafe sanitation (%)
C	43	Water	−0.244***	0.027	−0.018	0.000	−0.164	−0.076	Indicator 6.1.1: risk-weighted prevalence of populations using unsafe or unimproved water sources, as measured by the SEV for unsafe water (%)
D	12	HH Air Poll	−0.463***	0.041	−0.028	−0.007	−0.337	−0.070	Indicator 7.1.2: risk-weighted prevalence of household air pollution, as measured by the SEV for household air pollution (%)
E	29	Occ Burden	−0.014**	−0.002	−0.009	0.003	−0.001	−0.004	Indicator 8.8.1: age-standardised all-cause disability-adjusted life-year rates attributable to occupational risks (per 100 000 population)
F	24	Mean PM2.5	0.047***	0.035	−0.021	−0.033	−0.021	0.082	Indicator 11.6.2: population-weighted mean levels of fine particulate matter smaller than 2.5 μm in diameter (PM2.5)
G	6	Child Sex Abuse	−0.031***	0.005	−0.044	−0.007	0.004	0.012	Indicator 16.2.3: age-standardised prevalence of women and men aged 18–29 years who experienced sexual violence by age 18 (%)
G	34	Sexual Violence	0.005**	−0.006	0.007	0.006	0.003	−0.005	Indicator 16.1.3c: age-standardised prevalence of sexual violence experienced by populations in the past 12 months (%)
H	4	Cert Death Reg	0.048***	−0.001	−0.003	0.001	0.013	−0.003	Indicator 17.19.2c: Percentage of well-certified deaths by a vital registration system among a country’s total population (%)
J	5	Child Overweight	0.084***	0.001	0.017	0.006	0.033	0.003	Indicator 2.2.2b: prevalence of overweight in children aged 2–4 (%)
J	7	Child Stunting	−0.137***	0.071	−0.042	−0.102	−0.085	0.036	Indicator 2.2.1: prevalence of stunting in children under 5 years (%)
J	8	Child Wasting	−0.035*	0.005	−0.031	−0.010	0.047	−0.056	Indicator 2.2.2a: prevalence of wasting in children under 5 years (%)

***p<0.01, **p<0.05, *p<0.1.

NCD, non-communicable disease.

In the first column of table 2, CI for health-related SDGs indicators in 2017 (from table 1) are showed, and the first row shows the CI for the explanatory variables. Higher values of per capita GDP and per capita PHE were concentrated among more-developed states according to their SDI, as indicated by the positive CI values; on the contrary, health expenditures as percentage of GDP, illiteracy, and poverty were concentrated (as expected) among less-developed states. Overall, illiteracy and poverty had the strongest association with state-level inequalities for these HRSDGIs, highlighting the importance of these social determinants of health on inequalities. In contrast, PHE was not associated with these measures of health inequality.

Based on the sign for CI estimates for each health indicator and the sign of absolute contributions, overall larger values of GDP per capita was associated with decreasing inequalities when CI estimates were negative. The opposite occurred with higher levels of per capita health expenditure, illiteracy, and poverty, such that higher levels were associated with increased inequalities. Details for all indicators analysed are reported in online supplemental appendix, section 4.

## Discussion

Our analysis shows that Mexico is currently off track to meet the HRSDGIs by 2030,^19^ and persistent health inequalities across states may contributing to these trends. Based on our CI estimations and decomposition analysis, socioeconomic factors were associated with several HRSDGIs that demonstrated evidence of inequalities across Mexican states. These results emphasise the role of social determinants of health in Mexico, especially poverty and illiteracy.

Mexico’s inequalities in health have, in many ways, decreased from 1990 to 2017; however, for other health indicators, they have actually grown in magnitude. Mexico’s government sought to achieve the millennium development goals from 2000 to 2015, and increasingly is focused on the SDGs and particularly health indicators.^32 33^ These results are consistent with previous analyses based on data from the National Health Surveys 2006 and 2012; while there were gains in reducing inequalities in financial protection, such reductions were not evident for indicators on prenatal care and preventing certain non-communicable diseases (NCDs).^34^ Such findings are also consistent with previous studies on how place of residence is related to health outcomes and can promulgate ongoing inequalities.^14^

For Mexico, the only goal that was not achieved was that for maternal mortality (ie, a three-quarters reduction from 1990 to 2015). Despite these efforts, the current trends in health-related SDGs indicators reported here suggest that while most of them show progress, additional efforts are still required to close persistent gaps and be on track to meet the 2030 goals. This scenario worsens when considering country’s combination of historical social inequalities and lack of equity in resource allocation among states. In this analysis, we found that estimates on the magnitude and direction of inequalities were similar across socioeconomic stratifiers used (ie, SDI from the GBD and MI). We also compared those obtained from using percentage of individuals living in poverty and obtained similar results (not reported). In Mexico, as in most low-income and middle-income countries, increased investment is needed to address NCDs and tackle the very complex—and potentially compounding—effects of inequality, poverty and unhealthy behaviours.^35 36^ Without such action, making progress toward the SDGs, particularly among those who are greatest risk of being left behind, is unlikely. In this context, raising taxes on unhealthy products is one of the few mechanisms that could benefit the population and particularly the poor, making it a double-duty policy.^37^

Current government stakeholders must prioritise monitoring and evaluation of SDGs progress to prevent losing momentum toward 2030 goal attainment. Focus on identifying and closing gaps could help with directing strategies and regain momentum within the agenda. While there is evidence of progress in closing inequalities, as some gaps have been significantly reduced, health inequalities persist in Mexico. Most are related to social determinants of health, as the analysis suggests. This signals the need for a multisectoral approach focusing on social structural conditions and prioritising investment in the most vulnerable regions and populations, such as the southern region of the country, which exhibits a persistent lack of investment linked to divergent development progress.^38^ The fact that public resources devoted to health seem to have little impact on health inequalities suggests that allocation is currently not used as a mechanism to close gaps, but is still following a regressive historical budgetary pattern.^11 39 40^ Mexico’s health system’s fragmentation into subsystems and the subsequent segmentation of the population according to labour force participation has clearly contributed to the persistence of inequalities. As the system delivers lower quality services for those that are at the same time socioeconomically vulnerable, health services are in practice reproducing health inequalities.^8 11 34^

Advancing towards the closure of gaps on health indicators is certainly not a task only for the health sector, as health is socially determined and living conditions, education, among other factors play a key role. Increasing public investment in health is needed as out-of-pocket expenditure still comprises half of Mexico’s total health expenditure. However, this on its own is not enough as health requires to be part of a broader social protection scheme that provides reliable access to adequate housing, education, food and healthy environments for all.^28 41 42^ Inequalities in health and socioeconomic variables are well documented; however, the causal relationships between them are not easy to identify. This paper measures stronger statistical relationships between inequality in illiteracy and poverty and health, than between per capita GDP and health expenditure. This is not surprising if we contemplate that health variables affect human capital production and labour productivity, and more so in poorer populations. In addition, some ‘health’ indexes are moving in a direction opposite from the path marked by the SDGs, namely, those related to violence, which confirms the complex relationships between society and health. SDGs-related policies require a better understanding of these causal relationships and to identify useful levers for public policy. While the issues are complex, an effort must be made to assemble longitudinal databases to improve estimates and research, and to apply methods to control mutual causality and heterogeneity.

There are limitations that should be highlighted. It is possible that the information on some of the SDGs indicators, such as live births, HIV incidence, deaths due to interpersonal violence, maternal deaths, neonatal deaths or deaths of children under 5 years of age, may be underestimated. However, in each iteration, the GBD reanalyses the entire time series using newly available data and improved estimation methods, trying to diminish potential biases.^18 22^ The analysis was implemented at state level and therefore might overlook the relevant heterogeneity within states that could be even larger than between states. The National Observatory of Health Inequalities recently reported, for example, a CI of −0.21 for the infant mortality rate at the municipality level when stratified by poverty.^43^ The decomposition analysis used only a small number of variables that are available at the state level and could be relevant. More exploration is needed to include other variables. Also, the indicators included are not all relevant; for example, currently there is no state-level measure for catastrophic health spending. Unlike many countries, Mexico has a well-prepared information system to respond to international commitments of this importance in a timely manner and to meet disaggregation requests at the subnational level. In fact, in the national strategy^44^ and in the 2018 voluntary report,^32^ Mexico undertakes to follow 14 of the HRSDGI proposed at the international level and adds 13 more indicators for follow-up by 2030. All of them will be reported at the state level and two at the municipal level. Out of the 27 HRSDGI, 14 are reported by the Ministry of Health, 5 by Conapo, 5 by Inegi and 1 by each of the following institutions: Ministry of the Interior, Ministry of Labor and the Mexican Agency for Cooperation and International Development.

## Conclusion

Mexico’s performance on health-related SDGs indicators, while showing a positive trend as conditions are overall improving, is not enough to reach the 2030 commitments. In terms of intra-country progress, as inequalities are decreasing but persisting, there is a need for stronger pro-poor/pro-vulnerable populations policies focused on closing remaining gaps, allowing for national development while leaving no one behind.
