# Supplementary material for: Advances and challenges on the path toward the SDGs: subnational inequalities in Mexico, 1990–2017
Source: BMJ Glob Health. 2020 Oct 29;5(10):e002382. doi: 10.1136/bmjgh-2020-002382 (PMC7597504; doi:10.1136/bmjgh-2020-002382)
Supplement: Supplementary data [file bmjgh-2020-002382supp001.pdf]

Appendix to Advances and challenges in the path toward reaching the SDG in Mexico: inequalities and their decomposition by states, 1990–2017

1. Heatmaps: HRSDGI levels by Mexican states, 1990 and 2017 2
2. Trends of health-related SDG indicators in Mexico 3
3. Results of the concentration index by indicator... 4
4. Decomposition analysis by indicator... 8

Gutierrez JP, *et al. BMJ Global Health* 2020; 5:e002382. doi: 10.1136/bmjgh-2020-002382

1. Heatmaps: HRSDGI levels by Mexican states, 1990 and 2017

Indicators unscaled

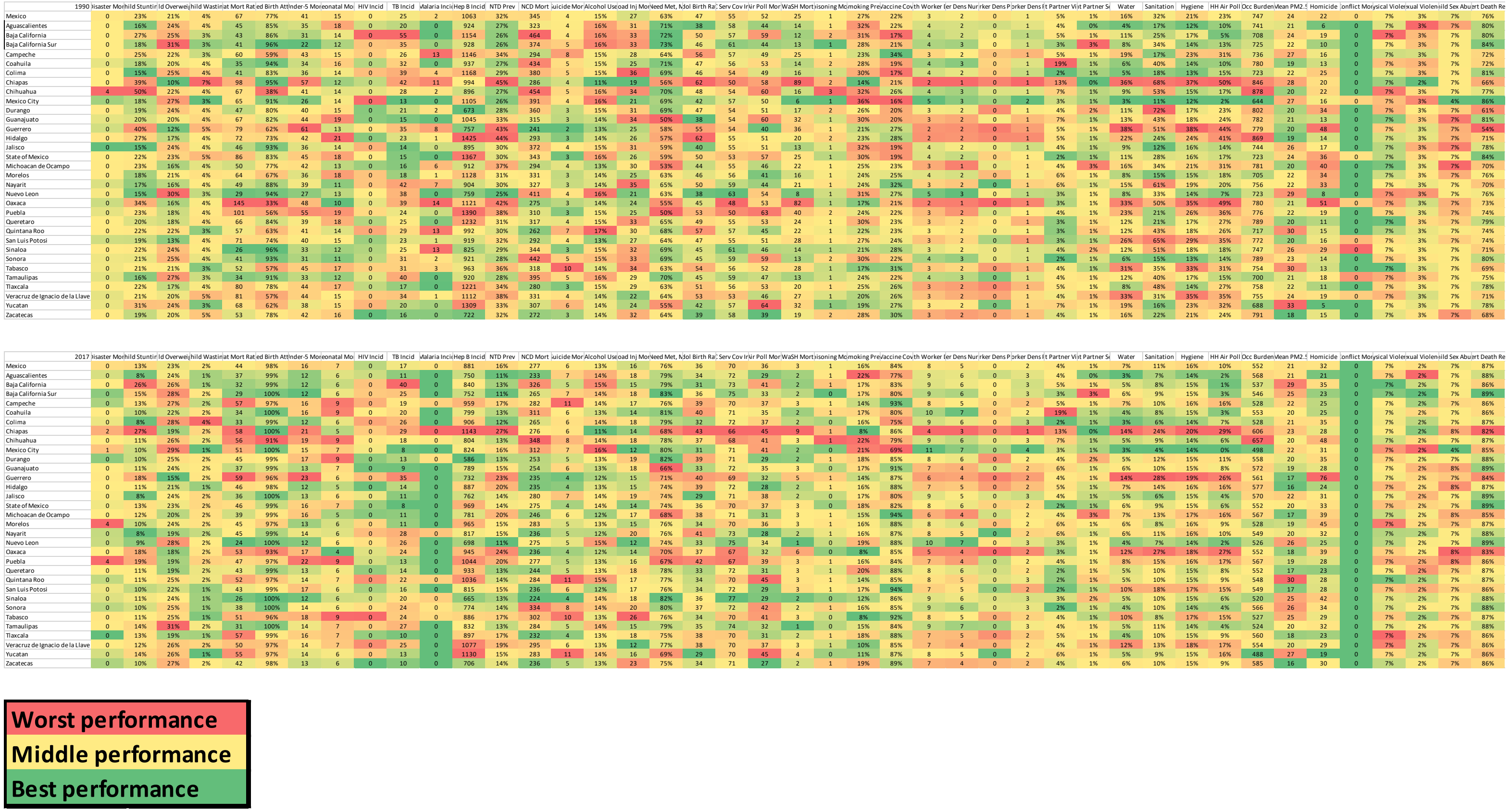

Scaled indicators

1990	SDG Index	isaster Mor	hild Stuntin	ild Overweig	hild Wastin	at Mort Rati	ed Birth Att	nder-5 Mor	eonatal Mor
Mexico	46	45	61	56	81	44	70	34	31
Aguascalientes	48	63	74	47	81	52	81	38	27
Baja California	44	60	54	44	85	53	81	41	34
Baja California Sur	45	58	71	29	87	54	95	49	38
Campeche	48	64	57	53	85	46	45	33	32
Coahuila	47	66	70	57	83	57	93	39	30
Colima	44	64	77	44	79	54	78	38	33
Chiapas	37	64	33	85	65	36	94	26	39
Chihuahua	34	0	12	52	81	44	18	34	34
Mexico City	43	71	70	39	89	44	88	45	33
Durango	40	52	69	48	81	51	73	35	31
Guanajuato	45	66	67	57	80	44	77	33	25
Guerrero	38	67	30	81	76	40	50	25	35
Hidalgo	42	42	55	67	82	42	64	34	22
Jalisco	46	79	77	48	83	52	91	37	34
State of Mexico	44	62	63	50	76	39	78	32	26
Michoacan de Ocampo	45	51	62	71	84	50	69	34	37
Morelos	48	50	71	55	81	45	56	37	26
Nayarit	49	67	73	68	83	50	85	36	40
Nuevo Leon	51	65	75	29	85	61	93	44	37
Oaxaca	34	68	41	70	83	28	12	31	44
Puebla	45	66	61	65	82	35	41	27	25
Queretaro	47	62	67	64	82	44	79	35	27
Quintana Roo	46	54	63	53	87	47	51	34	35
San Luis Potosi	47	67	68	76	82	43	65	35	33
Sinaloa	50	57	63	46	83	63	95	40	39
Sonora	48	65	66	44	83	54	91	41	41
Tabasco	45	65	64	56	87	49	43	32	28
Tamaulipas	50	66	74	38	85	57	88	40	39
Tlaxcala	44	64	64	66	83	40	71	32	28
Veracruz de Ignacio de la Llave	46	66	64	58	76	40	42	33	33
Yucatan	50	66	48	47	87	44	49	36	31
Zacatecas	47	52	69	57	78	48	71	34	30

1990	HIV Incid	TB Incid	Malaria Incid	Hep B Incid	NTD Prev	NCD Mort	uicide Mort	Alcohol Use	oad Inj Mor	Need Met, M	dol Birth Ra	Serv Cov In	Air Poll Mort	WaSH Mort	isoning Mo	moking Pre	Vaccine Cov
Mexico	45	69	30	58	67	60	87	53	26	65	15	39	41	27	31	28	9
Aguascalientes	47	73	57	63	73	64	87	50	21	76	21	44	47	34	28	10	9
Baja California	36	54	82	56	73	43	84	49	19	77	15	43	37	37	18	16	2
Baja California Sur	41	62	43	63	73	56	74	48	18	78	16	49	47	36	50	27	7
Campeche	42	68	19	56	65	70	59	53	24	67	10	43	43	27	29	44	23
Coahuila	49	64	56	63	72	47	80	54	29	75	16	42	41	34	24	26	5
Colima	41	60	27	55	71	55	77	53	16	73	16	38	43	33	35	17	3
Chiapas	48	59	21	61	54	72	91	64	39	56	7	32	38	10	16	72	7
Chihuahua	46	67	32	64	72	44	78	50	17	75	16	39	37	33	3	11	13
Mexico City	36	82	68	57	73	53	82	48	34	72	20	44	42	46	49	1	2
Durango	51	72	32	74	71	58	93	53	21	73	15	39	42	31	21	32	6
Guanajuato	51	79	57	59	66	66	94	56	17	49	21	39	37	23	34	17	6
Guerrero	44	62	22	70	56	82	100	59	29	59	9	38	49	22	28	45	14
Hidalgo	51	71	36	48	55	70	99	56	27	58	6	41	42	30	23	40	16
Jalisco	40	79	40	64	69	56	85	52	20	61	20	40	42	35	33	12	5
State of Mexico	45	78	57	50	70	61	100	50	27	60	14	38	39	27	37	17	5
Michoacan de Ocampo	50	78	25	64	62	70	90	58	22	52	17	40	45	28	30	33	10
Morelos	41	76	37	56	69	63	93	55	29	66	16	41	49	32	31	37	12
Nayarit	41	59	23	64	69	64	93	55	16	68	13	46	47	29	27	38	21
Nuevo Leon	45	61	61	70	75	50	83	48	35	66	22	53	40	41	35	16	14
Oaxaca	46	61	19	57	57	74	94	56	30	54	14	29	40	11	37	61	7
Puebla	43	70	40	49	61	67	100	54	28	49	11	33	36	20	27	36	9
Queretaro	49	69	46	53	68	65	89	53	18	68	13	41	40	27	31	18	10
Quintana Roo	39	66	20	61	70	77	64	47	22	71	10	43	46	29	31	46	10
San Luis Potosi	50	70	39	63	67	70	84	60	25	66	14	41	42	25	29	29	11
Sinaloa	49	69	19	67	70	61	99	52	19	72	16	50	46	34	37	48	16
Sonora	48	65	32	63	71	46	74	51	19	73	17	46	38	35	25	20	8
Tabasco	43	64	29	62	64	65	46	55	18	65	11	42	41	25	36	61	19
Tamaulipas	46	60	59	63	72	52	78	50	23	75	17	46	44	36	35	38	8
Tlaxcala	48	76	100	54	65	73	100	53	23	66	12	41	41	29	27	35	14
Veracruz de Ignacio de la Llave	42	63	37	57	62	63	81	55	33	67	12	38	45	26	34	51	13
Yucatan	40	73	42	51	66	67	66	54	29	55	18	43	35	24	43	53	14
Zacatecas	54	78	46	71	67	75	97	55	19	66	20	45	51	30	22	23	18

1990	th Worker D	t Partner Vi	nt Partner Se	Water	Sanitation	Hygiene	Air Poll Mort	Occ Burden	Mean PM2.5	Homicide	onflict Mor	ysical Violen	xual Violen	ild Sex Abu	ert Death Re
Mexico	51	97	75	82	65	80	41	51	50	12	65	72	51	73	82
Aguascalientes	56	99	88	96	82	90	47	52	56	42	100	72	45	72	87
Baja California	54	96	70	87	74	85	37	55	50	16	100	72	48	72	87
Baja California Sur	53	100	2	91	63	88	47	53	54	31	100	72	49	72	91
Campeche	43	96	75	78	82	78	43	52	50	21	100	72	55	72	78
Coahuila	50	48	84	93	56	88	41	49	60	25	100	72	48	72	78
Colima	47	100	86	95	81	89	43	54	55	10	100	72	49	72	88
Chiapas	6	66	91	58	25	63	38	44	46	15	100	72	69	72	71
Chihuahua	56	89	84	89	43	87	37	41	59	13	100	72	50	72	84
Mexico City	63	100	85	97	89	90	42	61	45	20	61	72	46	86	93
Durango	17	98	44	87	21	85	42	47	60	3	100	72	51	72	66
Guanajuato	49	88	82	85	53	83	37	49	57	25	100	72	49	72	88
Guerrero	3	97	71	56	44	62	49	49	58	0	100	72	54	72	59
Hidalgo	12	95	82	75	74	77	42	42	59	24	100	72	53	72	77
Jalisco	53	99	83	89	88	86	42	52	50	19	100	72	48	72	84
State of Mexico	48	100	85	87	69	85	39	54	50	2	69	72	50	72	91
Michoacan de Ocampo	49	99	16	82	63	80	45	49	58	0	100	72	52	71	75
Morelos	42	93	78	91	84	86	49	55	52	2	100	72	50	72	82
Nayarit	58	91	81	83	33	82	47	51	55	3	100	72	53	72	75
Nuevo Leon	56	100	79	91	64	88	40	54	45	38	100	72	47	72	82
Oaxaca	0	100	79	62	46	65	40	49	56	0	58	72	56	72	79
Puebla	51	99	79	74	78	75	36	49	54	16	100	72	53	72	80
Queretaro	49	100	71	86	78	84	40	48	58	29	100	72	50	72	86
Quintana Roo	51	100	59	86	53	84	46	54	46	22	100	72	53	72	80
San Luis Potosi	54	100	83	70	29	71	42	49	59	21	55	72	52	72	80
Sinaloa	53	100	51	86	44	84	46	52	50	6	46	72	51	72	77
Sonora	55	100	81	94	84	89	38	48	53	23	100	72	50	72	86
Tabasco	27	99	84	64	62	68	41	51	45	24	100	72	53	72	75
Tamaulipas	61	97	74	86	57	84	44	56	55	17	50	72	49	72	82
Tlaxcala	13	96	79	91	48	87	41	51	54	30	100	72	51	72	85
Veracruz de Ignacio de la Llave	32	99	54	62	66	66	45	51	52	16	59	72	55	72	77
Yucatan	58	89	71	78	83	79	35	57	44	49	100	72	53	72	84
Zacatecas	32	100	78	81	76	80	51	48	64	22	100	72	50	72	73

2017	SDG Index	isaster Mor	hild Stuntin	ild Overweig	hild Wastin	at Mort Rati	ed Birth Att	nder-5 Mor	eonatal Mor
Mexico	64	30	79	49	94	52	98	57	54
Aguascalientes	68	80	89	48	96	56	99	63	58
Baja California	66	80	57	41	96	59	99	63	60
Baja California Sur	62	80	76	34	94	61	100	64	57
Campeche	66	80	81	39	95	47	96	57	47
Coahuila	68	80	85	53	93	58	100	57	47
Colima	67	80	89	36	81	59	99	63	57
Chiapas	56	6	54	61	92	47	100	50	61
Chihuahua	63	80	84	42	94	47	89	53	47
Mexico City	62	13	85	33	97	49	100	58	53
Durango	65	80	85	45	94	52	99	56	46
Guanajuato	69	80	84	48	94	56	99	62	55
Guerrero	63	50	71	72	94	46	95	48	55
Hidalgo	69	80	83	54	96	51	98	63	60
Jalisco	68	80	89	47	92	56	100	62	55
State of Mexico	68	80	81	49	92	51	99	57	53
Michoacan de Ocampo	65	80	81	58	93	55	98	56	62
Morelos	55	0	85	48	93	52	97	63	59
Nayarit	68	80	89	60	95	52	99	60	58
Nuevo Leon	70	80	87	35	94	65	100	63	56
Oaxaca	56	68	70	64	93	48	91	55	68
Puebla	54	0	68	61	91	51	97	49	46
Queretaro	69	80	83	62	95	53	99	61	57
Quintana Roo	65	80	83	45	94	49	96	60	55
San Luis Potosi	69	80	86	54	96	53	99	56	57
Sinaloa	69	80	84	47	96	63	100	64	59
Sonora	66	80	85	43	96	55	100	60	56
Tabasco	63	44	83	45	97	50	95	54	46
Tamaulipas	68	59	79	29	94	60	100	60	52
Tlaxcala	68	80	81	61	96	47	99	57	53
Veracruz de Ignacio de la Llave	67	80	81	42	92	50	96	59	53
Yucatan	68	80	78	42	97	48	96	61	57
Zacatecas	67	80	85	39	94	53	98	61	59

2017	HIV Incid	TB Incid	Malaria Incid	Hep B Incid	NTD Prev	NCD Mort	uicide Mort	Alcohol Use	oad Inj Mor	Need Met, M	dol Birth Ra	Serv Cov In	Air Poll Mort	WaSH Mort	isoning Mo	moking Pre	Vaccine Cov
Mexico	50	76	65	65	83	73	70	58	46	82	23	64	53	56	55	64	84
Aguascalientes	49	85	100	70	88	84	64	54	40	86	24	66	60	61	52	43	75
Baja California	42	60	100	66	87	64	77	52	47	86	28	67	49	62	44	62	82
Baja California Sur	43	69	100	70	88	76	64	55	40	90	23	71	55	60	72	62	79
Campeche	41	74	61	62	83	72	44	57	44	82	21	64	52	56	53	72	95
Coahuila	47	73	100	68	87	67	71	60	49	88	20	65	54	61	48	60	79
Colima	40	68	100	64	88	76	68	56	41	86	26	65	52	60	62	64	73
Chiapas	43	66	61	56	73	74	69	66	50	72	18	57	45	40	41	89	86
Chihuahua	47	75	87	68	86	60	58	56	40	84	23	60	49	57	28	45	77
Mexico City	48	89	100	67	84	66	64	50	54	87	28	65	49	62	67	47	66
Durango	51	81	72	78	87	79	76	57	38	90	21	64	60	62	49	59	85
Guanajuato	53	89	100	68	84	78	66	60	41	69	26	66	54	57	60	60	92
Guerrero	42	63	100	71	76	83	89	62	47	75	20	61	57	49	46	72	87
Hidalgo	53	80	100	64	80	83	85	59	46	79	21	65	60	60	52	63	88
Jalisco	48	85	100	69	86	73	61	57	39	79	29	65	52	59	59	62	79
State of Mexico	51	91	100	61	85	74	81	57	51	80	23	62	52	56	64	58	81
Michoacan de Ocampo	52	85	100	69	80	81	72	63	42	71	22	65	58	57	53	69	96
Morelos	44	84	100	62	85	72	75	58	47	82	25	63	52	57	51	63	89
Nayarit	42	67	84	67	85	83	78	61	37	82	20	67	61	60	56	64	87
Nuevo Leon	46	68	100	72	89	74	74	53	54	80	26	71	55	65	59	56	88
Oaxaca	48	70	100	62	76	83	82	61	49	74	22	59	56	46	65	89	86
Puebla	49	81	100	59	80	73	78	59	44	71	20	58	50	53	51	62	84
Queretaro	51	79	100	63	87	81	76	58	40	84	26	65	57	56	57	51	89
Quintana Roo	38	71	66	59	86	72	44	53	42	83	25	63	46	55	53	71	85
San Luis Potosi	50	78	100	67	84	83	66	62	43	82	25	65	60	54	54	61	96
Sinaloa	49	73	74	74	87	86	91	56	41	90	23	73	60	64	64	77	87
Sonora	46	70	67	69	86	62	56	55	37	87	23	67	48	58	47	65	86
Tabasco	36	70	64	64	83	68	46	58	27	81	25	63	49	56	58	90	93
Tamaulipas	43	67	100	67	86	72	76	55	47	85	24	69	57	65	61	68	83
Tlaxcala	51	87	100	64	83	84	85	57	41	80	22	63	57	59	50	58	89
Veracruz de Ignacio de la Llave	38	69	100	58	81	70	71	58	55	83	22	63	52	57	58	84	85
Yucatan	43	81	100	56	85	72	45	56	46	73	30	63	46	53	63	79	87
Zacatecas	54	86	100	72	86	83	79	59	32	81	24	65	62	59	44	55	90

2017	th Worker D	t Partner Vi	nt Partner Se	Water	Sanitation	Hygiene	Air Poll Mort	Occ Burden	Mean PM2.5	Homicide	onflict Mor	ysical Violen	xual Violen	ild Sex Abu	ert Death Re
Mexico	78	97	75	92	88	86	53	71	55	4	100	71	71	71	94
Aguascalientes	82	99	88	97	93	88	60	69	58	14	100	71	69	70	95
Baja California	78	96	70	94	92	87	49	72	46	2	100	72	72	71	93
Baja California Sur	79	100	1	93	90	87	55	71	51	12	100	72	74	71	97
Campeche	74	97	75	92	90	86	52	74	58	9	100	71	72	70	93
Coahuila	76	47	83	95	92	87	54	71	59	9	100	71	71	71	93
Colima	77	100	85	96	94	88	52	74	57	2	100	71	72	71	94
Chiapas	51	67	91	84	74	82	45	65	54	7	100	71	73	70	89
Chihuahua	80	89	84	95	91	87	49	60	59	0	100	72	71	71	94
Mexico City	86	100	85	97	96	88	49	77	53	4	100	71	69	86	92
Durango	63	99	43	94	87	86	60	70	62	2	100	71	71	71	95
Guanajuato	77	92	80	93	90	86	54	68	61	7	100	71	70	70	96
Guerrero	50	98	71	84	69	82	57	70	65	0	100	71	71	70	91
Hidalgo	65	96	81	92	86	86	60	68	64	10	100	71	71	70	94
Jalisco	81	99	83	95	94	87	52	69	55	5	100	71	70	71	96
State of Mexico	75	100	85	94	91	87	52	71	56	3	100	71	71	70	96
Michoacan de Ocampo	77	99	15	92	87	85	58	69	65	0	100	72	71	70	92
Morelos	73	93	78	93	92	86	52	74	59	0	100	71	71	70	94
Nayarit	84	92	81	93	88	86	61	71	60	4	100	72	72	71	95
Nuevo Leon	79	100	79	96	93	88	55	74	49	9	100	72	71	71	96
Oaxaca	4	100	78	86	71	83	56	71	64	0	100	71	71	70	90
Puebla	79	100	79	91	85	86	50	69	59	7	100	71	70	70	93
Queretaro	80	100	71	94	90	87	57	71	63	12	100	71	70	70	94
Quintana Roo	79	100	59	94	90	87	46	71	46	7	100	72	72	71	94
San Luis Potosi	81	100	83	89	81	84	60	71	65	7	100	71	70	70	93
Sinaloa	81	100	51	94	90	87	60	75	52	0	100	71	72	71	95
Sonora	81	100	81	95	90	88	48	69	50	3	100	72	71	71	96
Tabasco	69	100	84	88	92	84	49	74	52	6	100	71	72	70	94
Tamaulipas	84	97	74	95	89	87	57	74	58	4	100	71	71	71	95
Tlaxcala	60	96	79	96	89	87	57	70	62	11	100	71	70	70	93
Veracruz de Ignacio de la Llave	69	99	53	87	87	84	52	71	60	6	100	71	72	70	93
Yucatan	85	93	71	95	91	87	46	79	52	16	100	71	72	71	93
Zacatecas	68	100	78	93	89	86	62	67	68	6	100	72	71	70	93

Worst performance
Middle performance
Best performance

Gutierrez JP, *et al. BMJ Global Health* 2020; 5:e002382. doi: 10.1136/bmjgh-2020-002382

2. Trends of health-related SDG indicators in Mexico

From the 40 analysed indicators, the trend for 22 is in the desired direction, that is, to improve conditions among the population (maternal mortality ratio [MMR], births attended by skilled personnel, under-5 mortality, neonatal mortality, malaria incidence, neglected tropical diseases [NTD] prevalence, road injuries mortality, met need for family planning, adolescent birth rate, coverage of essential health services, air pollution mortality, unsafe water, sanitation or lack of hygiene [WaSH] mortality, unintentional poisoning mortality, health worker density, unsafe water, unsafe sanitation, access to handwashing facility, prevalence of household air pollution, occupational risks, well-certified deaths, stunting, wasting). Of the remaining 21, there were no changes or very limited changes in recent years for 16, so almost a stable pattern (tuberculosis incidence, hepatitis incidence, non-communicable diseases mortality, self-harm mortality, alcohol consumption, prevalence of daily smoking, vaccine coverage, intimate partner violence, non-intimate partner violence, levels of $PM_{2.5}$, conflict mortality, physical violence, sexual violence, childhood sexual abuse, forces of nature mortality, child overweight) while for the last two, the trend is in the opposite direction (HIV incidence and interpersonal violence mortality).

3. Results of the concentration index by indicator

Of a total of 25 indicators concerning SDG, 18 presented evidence of inequalities. Fertility rate for women aged 10 to 19 consistently had a CI with negative values from 1990 up to 2017. Although CI values are relatively low (-0•048 p = 0•011 in 1990 to -0•043 p = 0•008 in 2017), what seems relevant is that inequality remains in the period, with a reduction between 1990 and 2017 of only about 10%.

For the death rate attributable to air pollution, incidence of HIV, neonatal mortality rate, death rate for unintentional poisoning, death rate due to road injuries, death rate due to self-harm, and TB incidence, there was no evidence of inequality across states in Mexico.

Prevalence of alcohol consumption was concentrated among states with higher development, with almost no changes between 1990 and 2017: the CI for the former year was 0•048 (p=0•006) and for the latter 0•047 (p=0•006), suggesting a stable pattern.

The percentage of women of reproductive age with met need for family planning was larger in more developed states, with negligible changes over the analysed period: the CI was 0•105 (p<0•001) in 1990 and 0•091 (p<0•001) in 2017.

Regarding the incidence of HIV, there was evidence of inequality only in 1990, with a CI of 0•112 (p = 0•003).

In terms of the indicators of health personnel, for the overall index as well as for each of the three types of personnel (nurses, physicians, and pharmacists), the CI values are positive (ie, personnel more concentrated in more-developed states) and statistically different from zero. The CI for the overall index, density of health workers, went from 0•144 (p<0•001) in 1990 to 0•111 (p<0•001) in 2017.

The rate of hepatitis incidence was slightly but increasingly concentrated in less-developed states during the period 1990–2017, with a CI of -0•015 (p=0•445) in 1990 and a CI of -0•045 (p=0•003) in 2017, representing a 200% increase in the CI value over the 27 years.

The inequality in the distribution of the rate of malaria was identified during the period with high values of the CI: it was -0•494 (p=0•004) in 1990 and increased to -0•652 (p=0•055) in 2017.

The CI values for the maternal mortality ratio (MMR) decreased 50%, from a CI of -0•122 (p=0•001) in 1990 to a CI in 2017 of -0•064 (p=0•002) in 2017 that was concomitant with an overall reduction of the MMR in the country.

The CI death rate due to non-communicable diseases for the population aged 30 to 70 has positive values for the period, but decreased from 0•075 (p<0•001) in 1990 to 0•029 (p=0•007) in 2017, that is, a 61% reduction.

As expected, prevalence of neglected tropical diseases is more concentrated among less-developed states. The CI for this indicator decreased 10% over the period, as it presented a value of -0•144 (p<0•001) in 1990 and then in 2017 a CI of -0•130 (p<0•001).

The proportion of births that are attended by skilled health personnel was higher in more-developed states, so the CI has positive values during the 1990–2017 period. The CI in 2017 was 0.263 (p=0•027), which represented a 9% decrease from the CI in 1990 of 0.288 (p=0•002).

Prevalence of current smoking was also higher in more-developed states, but with a decreasing CI during the analysed period. The CI for this indicator was 0•142 (p<0•001) in 1990 and was 0•110 (p<0•001) by 2017, a 23% reduction.

Coverage of essential health services was slightly concentrated among more-developed states during this period, with a decreasing pattern. The CI for this indicator went from 0•050 (p<0•001) in 1990 to 0•042 (p<0•001) in 2017, a 16% decrease.

The CI values for the under-5 mortality rate are negative in the analysed years – that is, more frequent among states with lower SDI values, with a decrease of 29% in the period: the CI in 1990 was -0•105 (p<0•001) and -0•075 (p<0•001) in 2017.

Interestingly, vaccine coverage is more concentrated among less-developed states with an increasing pattern: the CI for this indicator was -0•065 (p=0•006) in 1990 and more than doubled by 2017, with a CI of -0•148 (p=0•037).

The CI values for death rate attributable to unsafe water, sanitation, and hygiene (WaSH) are negative for the period, with a reduction of 35% from 1990 to 2017; that is, the CI in 1990 was -0•327 (p<0•001) and -0•211 (p<0•001) in 2017.

With regard to the two health-related indicators of SDG 5, gender equality, neither for the prevalence of women experiencing violence by an intimate partner in the last 12 months nor the prevalence experiencing violence by a non-intimate partner in the last 12 months there was evidence of inequality.

For SDG 6, clean water and sanitation, there are three health-related indicators, and for all of those, there was evidence of inequality affecting population living in less-developed states. Prevalence of population without access to a handwashing facility was concentrated in less-developed states, with a clear trend of reducing inequality. The CI for this indicator was -0•251 (p<0•001) in 1990 and decreased to -0•061 (p<0•001) in 2017, a 76% reduction in 27 years.

In an opposite trend, the inequality in prevalence of populations using unsafe or unimproved sanitation increased in the period in 24%. The CI for this indicator went from -0•213 (p=0•005) in 1990 to -0•267 (p<0•001) in 2017.

Finally, inequality in the prevalence of populations using unsafe or unimproved sources of water decreased in the period, although it was still relevant by 2017.

The CI for this indicator was -0•397 (p=0•005) in 1990 and dropped by 39% to -0•244 (p<0•001) in 2017.

For SDG 7, affordable and clean energy, there is one health-related indicator, prevalence of household air pollution, and there was evidence of increasing inequality for it, affecting less-developed states: the CI was -0•427 (p<0•001) in 1990 and increased to -0•463 (p<0•001) in 2017, that is, 8% higher.

All-cause disability-adjusted life year rates attributable to occupational risks is the health-related indicator for SDG 8, decent work and economic growth. The estimated CIs for the period indicate evidence of inequality that decreased over the period, with a CI of -0•027 (p<0•001) in 1990 and a CI of -0•014 (p=0•012) in 2017.

There is one health-related indicator for SDG 11, sustainable cities and communities, the mean levels of fine particulate matter smaller than 2.5 microns in diameter. The analysis indicates that this value is higher among more-developed states, with an increasing pattern. For this indicator, the CI was 0•027 (p=0•064) in 1990 and 0•047 (p=0•002) in 2017.

For SDG 16, peace, justice, and strong institutions, there are five health-related indicators, and for three of them (death rate due to conflict and terrorism, death rate due to interpersonal violence, and prevalence of physical violence), there is no evidence of inequalities.

On the other hand, for the indicator on child sexual abuse (prevalence of men and women aged 18-29 years that experienced sexual violence by the age 18), the CI values indicate a slight concentration among less-developed states, with the CI being -0•046 (p=0•001) in 1990 and -0•031 (p=0•007) in 2017.

The prevalence of sexual violence was, in turn, slightly concentrated in more-developed states during the period, with a decreasing trend in the CI values: the CI was 0•030 (p<0•001) in 1990 and 0•005 (p=0•018) in 2017.

For SDG 17, partnerships for the goals, there is one health-related indicator, the percentage of well-certified deaths. The CI for this indicator was 0•158 (p<0•001) in 1990 and dropped to 0•048 (p=0•003) in 2017.

The health-related indicator for SDG 1, no poverty, death rate due to exposure to forces of nature, presented no evidence of inequalities during the period; that is, the CI were not statistically different from zero.

Finally, three health-related indictors were included for SDG 2, zero hunger, and for all three there was evidence of inequality. Regarding overweight among children aged 2 to 4, there was an important reduction in the CI absolute value, from 0•148 (p < 0•001) in 1990 to 0•084 (p < 0•001) in 2017, equivalent to 46% decline. In this case, the positive value indicates that child overweight is more frequent in more-developed states, but the trend is to equalise across the SDI. With regard to stunting and wasting, for both indicators (prevalence in children under 5), the CI values indicate inequalities, with higher prevalence among less-developed states. While for wasting there is a decreasing trend in inequality, for stunting the inequality increased from 1990 to 2017. The CI for prevalence of stunting was -0•118 (p=0•008) in 1990 and increased to -0•137 (p=0•001) in 2017. For wasting prevalence, the CI went from -0•070 (p=0•002) in 1990 to -0•035 (p=0•084) in 2017, that is, almost negligible change.

4. Decomposition analysis by indicator

For inequality on the adolescent fertility rate, while GDP per capita seems to reduce it (opposite sign, absolute contribution of 0•030), the larger contributor to it is poverty, with an absolute contribution of -0•036. For alcohol use, the larger contributor is illiteracy, with an absolute contribution of 0•018; this indicator is also the larger contributor to the inequality in family planning: 0•013. For the inequality in the density of health care personnel, illiteracy is also the larger contributor, with an absolute contribution of 0•058, while poverty is the second contributor with 0•049.

For inequality on the incidence of hepatitis B, poverty is the larger contributor with an absolute contribution of -0•093, while for malaria incidence it is the public health expenditure percentage of GDP with an absolute contribution of -1•091.

For inequality in maternal mortality ratio, the largest and sign coincident contribution is from poverty, with a value of -0•098, while for mortality due to chronic conditions it is illiteracy, with 0•022. For inequality in the prevalence of

neglected tropical diseases, again illiteracy is the larger contributor with a value of -0•085.

For inequality in the prevalence of smoking, again illiteracy is the larger contributor, with a value of 0•090, while for inequality in the indicator for UHC, poverty is the larger contributor, with an absolute contribution of 0•019.

The largest contributor to inequality in under-5 mortality was poverty, with absolute contribution of -0•073, almost as large as the CI for the health indicator.

In turn, the largest value of absolute contribution to inequality in vaccine coverage is the per capita public health expenditure, with a value of -0•018.

For the inequality in WaSH mortality, public health expenditure as percentage of the state GDP is the explanatory variable, with the larger sign-coincident value of absolute contribution with -0•171. Inequalities in the prevalence of unsafe or unimproved handwashing facilities, sanitation, and water are explained principally by illiteracy, with values of absolute contribution of -0•038, -0•243, and -0•164, respectively. Illiteracy is also the variable that explains most of the inequality in the prevalence of household pollution, with an absolute contribution of -0•337.

Poverty turns out to be the variable with the larger explanation for inequality in the mean levels of $PM_{2•5}$, with an absolute contribution of 0•082.

Regarding inequality in child sex abuse, per capita public health expenditure is the variable that explains more of it, with an absolute contribution of -0•044, and this is also the variable that explains more of the inequality in sexual violence, with an absolute contribution of 0•007.

The inequality in well-registered deaths is explained mostly by illiteracy, with an absolute contribution of 0•013.

Illiteracy is also the variable with the larger value of absolute contribution to inequality in child overweight, 0•033. Regarding inequalities in the indicators of child malnourishment by deficiencies, per capita public health expenditure is the larger contributor to stunting and poverty to wasting, with absolute contributions of -0•102 and -0•056, respectively.

Gutierrez JP, *et al. BMJ Global Health* 2020; 5:e002382. doi: 10.1136/bmjgh-2020-002382
